# Hand factor ablation causes defective left ventricular chamber development and compromised adult cardiac function

**DOI:** 10.1371/journal.pgen.1006922

**Published:** 2017-07-21

**Authors:** Joshua W. Vincentz, Kevin P. Toolan, Wenjun Zhang, Anthony B. Firulli

**Affiliations:** 1 Department of Pediatrics, Riley Heart Research Center, Herman B Wells Center for Pediatric Research, Indiana University School of Medicine, Indianapolis, Indiana, United States of America; 2 Department of Anatomy & Cell Biology, Indiana University School of Medicine, Indianapolis, Indiana, United States of America; 3 Department of Medical & Molecular Genetics, Indiana University School of Medicine, Indianapolis, Indiana, United States of America; 4 Department of Biochemistry & Molecular Biology, Indiana University School of Medicine, Indianapolis, Indiana, United States of America; Weis Center for Research, Geisinger Clinic, UNITED STATES

## Abstract

Coordinated cardiomyocyte growth, differentiation, and morphogenesis are essential for heart formation. We demonstrate that the bHLH transcription factors Hand1 and Hand2 play critical regulatory roles for left ventricle (LV) cardiomyocyte proliferation and morphogenesis. Using an LV-specific *Cre* allele (*Hand1*^*LV*^*-Cre*), we ablate *Hand1*-lineage cardiomyocytes, revealing that DTA-mediated cardiomyocyte death results in a hypoplastic LV by E10.5. Once *Hand1*-linage cells are removed from the LV, and *Hand1* expression is switched off, embryonic hearts recover by E16.5. In contrast, conditional LV loss-of-function of both *Hand1* and *Hand2* results in aberrant trabeculation and thickened compact zone myocardium resulting from enhanced proliferation and a breakdown of compact zone/trabecular/ventricular septal identity. Surviving *Hand1*;*Hand2* mutants display diminished cardiac function that is rescued by concurrent ablation of *Hand*-null cardiomyocytes. Collectively, we conclude that, within a mixed cardiomyocyte population, removal of defective myocardium and replacement with healthy endogenous cardiomyocytes may provide an effective strategy for cardiac repair.

## Author summary

The left ventricle of the heart drives blood flow throughout the body. Impaired left ventricle function, associated either with heart failure or with certain, severe cardiac birth defects, constitutes a significant cause of mortality. Understanding how heart muscle grows is vital to developing improved treatments for these diseases. Unfortunately, genetic tools necessary to study the left ventricle have been lacking. Here we engineer the first mouse line to enable specific genetic study of the left ventricle. We show that, unlike in the adult heart, the embryonic left ventricle is remarkably tolerant of cell death, as remaining cells have the capacity to proliferate and to restore heart function. Conversely, disruption of two related genes, *Hand1* and *Hand2*, within the left ventricle causes cells to assume the wrong identity, and to consequently overgrow and impair cardiac function. Ablation of these mutant cells rescues heart function. We conclude that selective removal of defective heart muscle and replacement with healthy cells may provide an effective therapy to treat heart failure.

## Introduction

The left ventricle (LV) of the heart drives systemic circulation. Because the LV must be large enough to support adequate cardiac output but not hypertrophic or hypoplastic, such that it obstructs blood flow, congenital heart defects (CHDs) and acquired diseases that impact LV morphology or cell number present a significant cause of morbidity and mortality in the human population [1]. These CHDs include left ventricular noncompaction or hypertrabeculation (LVNC; OMIM: 604169), which is a cardiomyopathy characterized by prominent trabeculations occluding the ventricular lumen and associated with a high risk of heart failure and sudden death [2, 3] and hypoplastic left heart syndrome (HLHS; OMIM: 614435), which presents an underdeveloped LV unable to sustain sufficient blood flow [4, 5]. From the perspective of adult cardiac disease, especially cardiomyopathies, it is of particular importance to establish the capacity of proliferating cardiomyocytes to replace dead or dysfunctional cardiomyocytes [1]. Critical breakthroughs in patient outcomes demand a better understanding of the etiology of cardiac growth, differentiation and morphogenic patterning.

The embryonic heart forms from two distinct cardiomyocyte progenitor populations, termed the primary (PHF) and secondary (SHF) heart fields, which give rise to the LV and right ventricle (RV), respectively [6]. The bHLH transcription factor *Hand1* is predominantly expressed within the LV myocardium, with minimal expression in SHF derivatives [7]. Interestingly, *HAND1* mutations have been identified in HLHS patients [8]; however, cardiac-specific *Hand1* ablation in mice does not recapitulate HLHS [9]. Previous studies suggest that the related bHLH transcription factor Hand2 can functionally cooperate with Hand1 during murine LV development [9]. Although *Cre*-loxP technology has been used to great effect in mice to genetically model SHF myocardium defects, the genetic tools to specifically interrogate genetic loss-of-function in LV cardiomyocytes have, thus far, not been available.

We have isolated a conserved 744 bp enhancer 5’ to the *Hand1* transcription start site that is sufficient to drive reporter and *Cre recombinase* gene expression specifically within the LV. *Hand1*^*LV*^*-Cre* recapitulates endogenous *Hand1* expression within an estimated 80–90% of LV cardiomyocytes between embryonic stages (E) E8.5-E13.5. Ablation of the *Hand1*-lineage LV myocardium results in a markedly smaller LV by E10.5; however, LV chamber size and adult cardiac function is ultimately rescued via proliferation of non-*Hand1*-lineage LV cardiomyocytes.

In contrast to this LV cell ablation model, *Hand1*;*Hand2* loss-of-function within the LV results in increased cardiomyocyte proliferation resulting in morphogenic defects that lead to the occlusion of the LV lumen. LV cardiomyocytes invade the LV chamber and show mis-expression of compact zone, trabeculae, and intraventricular septum (IVS) restricted genes. We reveal a cooperative Hand factor function that is required to morphogenetically specify subpopulations of ventricular myocardium, and to thereby regulate cardiomyocyte growth. Functional analysis of surviving *Hand1*;*Hand2* double conditional knockouts (CKOs) reveals impaired LV function that can be rescued by ablating the mutant *Hand1* LV-lineage cells. Taken together, these data suggest that, due to its inherent proliferative capacity, the developing LV tolerates myocardial cell death, whereas developmentally abnormal cardiomyocytes adversely impact cardiac function.

## Results

### A distal *Hand1* enhancer recapitulates gene expression within the LV

**Fig 1:**
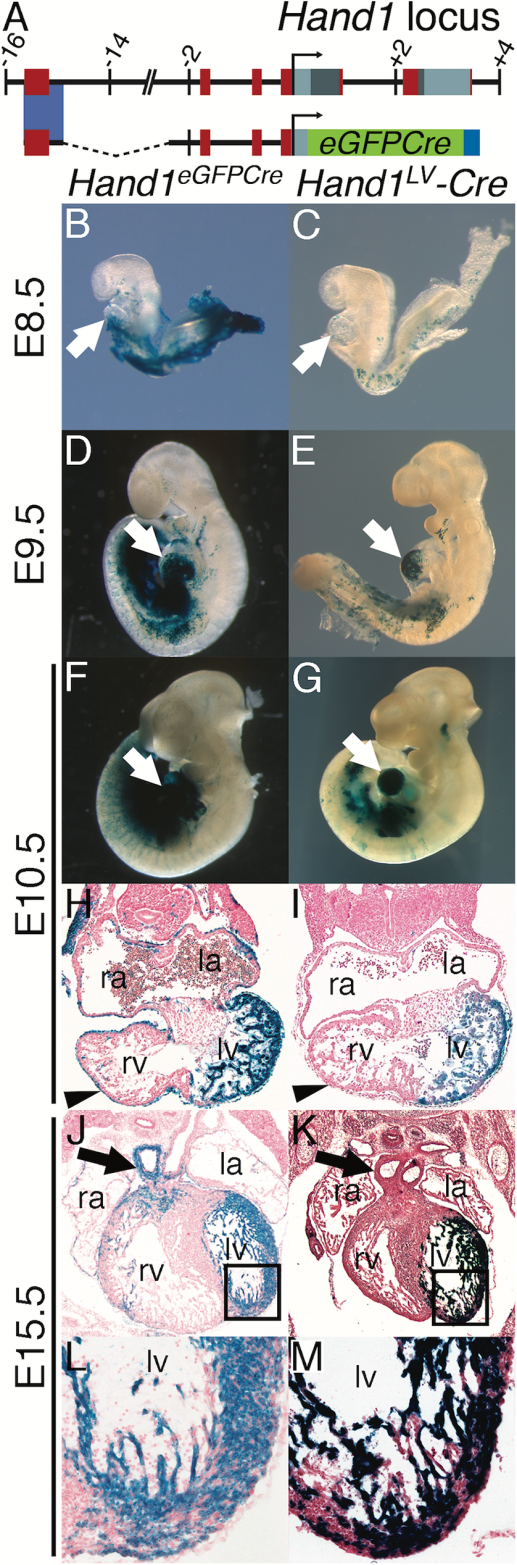
The *Hand1*^*LV*^*-Cre* transgenic allele recombines within the embryonic LV. A) Schematic of the mouse *Hand1*^*LV*^*-Cre* transgene, in which a 744bp *Hand1* LV-specific enhancer and ~2.7Kb *Hand1* endogenous promoter regulate an *eGFPCre* expression cassette. B-M) X-gal staining to detect the *R26R*^*lacZ*^ reporter allele demonstrates that, like the *Hand1*^*eGFPCre*^ knock-in allele (B, D, F, H, J, L), *Hand1*^*LV*^*-Cre* activity initiates in the LV (white arrows) between E8.5 and E9.5 (C, E). Cre-lineage cells are detectable within the LV at E10.5 (G, I) and E15.5 (K, M), but not within the epicardium (H, I; black arrowheads) or the outflow tract (H, I; black arrows). la–left atrium, ra–right atrium, lv–left ventricle, rv–right ventricle.

We have identified a *cis*-regulatory element(s) that drives gene expression specifically within the LV, and not within other cardiac tissues (Vincentz and Firulli, manuscript in preparation). We used this *Hand1* enhancer to make an LV-specific *Cre* driver. Lineage analysis using the *Hand1*^*eGFPCre*^ knock-in allele revealed that *Hand1*-lineage cells are restricted to the LV myocardium and to a ring of SHF-derived myocardium occupying the OFT, termed the myocardial cuff [7, 10]. Using the 2.7kb *Hand1* basal promoter to provide the *eGFPCre* with a transcriptional start site, we cloned the *Hand1* LV-enhancer 5’ (Fig 1A) and generated several F0 transgenic lines. In two of these lines, Cre activity, as assessed via the *R26R*^*lacZ*^ reporter allele, was detectable within the forming LV at E9.0 (Fig 1E; white arrow) specifically marking only the *Hand1* LV cardiomyocyte-lineage (Fig 1E, 1G, 1I, 1K and 1M). *Hand1*-lineage epicardium (black arrowhead; Fig 1H and 1I) and OFT tissues (Fig 1J and 1K; black arrows), detectable from the *Hand1*^*eGFPCre*^ allele, were not observed in the *Hand1*^*LV*^*-Cre* transgenic. Immunohistochemistry using β-galactosidase to mark Cre-lineage cells and Mlc2v to mark ventricular cardiomyocytes showed total co-localization of these two markers (S1A, S1C, S1E and S1G Fig), whereas expression of β-galactosidase and PECAM, an endothelial/endocardial marker, appears mutually exclusive (S1A, S1C, S1E and S1G Fig). Together, these data validate that this *Hand1*^*LV*^*-Cre* driver recombines specifically within LV cardiomyocytes.

### Cellular ablation of the *Hand1*^*LV*^*-Cre* embryonic LV myocardium results in hypoplastic LV at E10.5 that fully recovers in size and function

**Fig 2:**
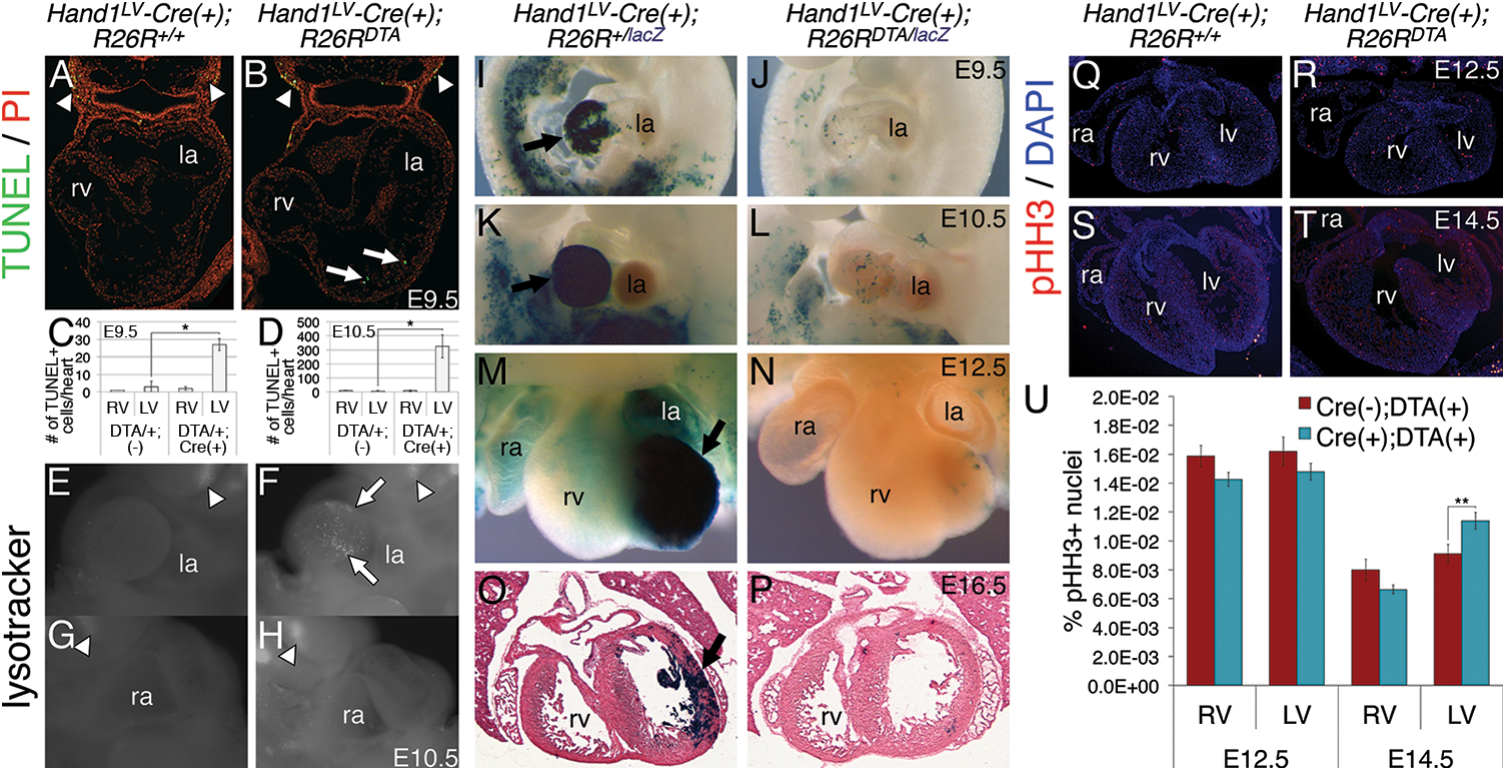
*Hand1*^*LV*^*-Cre*-mediated DTA activation results in LV cardiomyocyte cell death and hypoplastic LV, which, through cardiomyocyte proliferation, recovers fully by E16.5. A, B) TUNEL on E9.5 sections of *Hand1*^*LV*^*-Cre(+);R26R*^*lacZ/+*^ control (A) and *Hand1*^*LV*^*-Cre(+);R26R*^*lacZ/DTA*^ embryos. Sections are counterstained with propidium iodide (PI). C, D) Quantification of the number of TUNEL-positive cells per heart in control and *Hand1*^*LV*^*-Cre(+);R26R*^*lacZ/DTA*^ embryos at E9.5 (C) and E10.5 (D). E-H) Whole mount lysotracker staining detecting cell death confirms that conditional activation of *R26R*^*eGFPDTA/+*^ via intercross to *Hand1*^*LV*^*-Cre* causes pronounced LV cardiomyocyte death as assayed at E10.5 (white arrow; n = 3). White arrows denote TUNEL or lysotracker-positive cells in the LV. White arrowheads denote TUNEL or lysotracker-positive cells in the pharyngeal arches. I-P) X-Gal staining to detect the *R26R*^*lacZ*^ reporter allele demonstrates that activation of the *R26R*^*eGFPDTA*^ allele nearly completely ablates the *Hand1*^*LV*^*-Cre* lineage (black arrow) by E9.5 (I, J; n = 5); however, LV hypoplasia is not apparent until E10.5 (K, L; n = 3) and persists at E12.5 (M, N; n = 4). Despite DTA-mediated ablation of the *Hand1*^*LV*^*-Cre* lineage, by E16.5, the initially hypoplastic LV shows a pronounced recovery, and LV size is comparable to controls (O, P; n = 3). Q-T) Immunohistochemistry for pHH3 at E12.5 (Q, R) and E14.5 (S, T) in *Hand1*^*LV*^*-Cre(+);R26R*^*+/+*^ control (Q, S) and *Hand1*^*LV*^*-Cre(+);R26R*^*+/DTA*^ embryos (R, T). U) Quantification of pHH3+ cells relative to the number of DAPI+ pixels show that proliferation is not altered at E12.5, but is elevated specifically within the LV at E14.5. Data are represented as mean ± standard error of mean. Asterisks denote significance (p ≤ 0.05) as determined by student’s t-test.

To address the importance of the *Hand1*^*LV*^ lineage during cardiac development, we utilized the conditionally active *Rosa26 (R26R) Diphtheria Toxin A chain* (*R26R*^*DTA*^) allele to conditionally ablate *Hand1*-expressing LV cardiomyocytes. Analysis of cell death via TUNEL on E9.5 sections revealed that the LVs of *Hand1*^*LV*^*-Cre; R26R*^*lacZ/+*^ control embryos displayed few apoptotic cells (Fig 2A), whereas *Hand1*^*LV*^*-Cre; R26R*^*lacZ/DTA*^ LVs contained scattered TUNEL-positive cells (Fig 2B, quantified in Fig 2C). However, given the robust LV expression of the *Hand1*^*LV*^*-Cre*, fewer TUNEL-positive cells than would be predicted were detected in E9.5 *R26R*^*DTA*^-ablated LVs. Additional TUNEL staining, (Fig 2D) and whole mount lysotracker staining at E10.5 revealed markedly increased cell death within the LV of *Hand1*^*LV*^*-Cre; R26R*^*lacZ/DTA*^ embryos when compared to controls (white arrows Fig 2E and 2F). At both time points, no difference in cell death was observed in the RV myocardium (Fig 2A–2D, 2G and 2H), and cell death in the pharyngeal arches served as a positive control (white arrowheads Fig 2A, 2B and 2E–2H).

We then bred the *R26R*^*lacZ*^ reporter onto the *R26R*^*DTA*^ allele to monitor the loss of *Hand1*-expressing LV cardiomyocytes in our ablation model. At E9.5, the *Hand1*-LV lineage is largely absent from the heart, but the LV is not grossly hypoplastic (Fig 2J). By E10.5, the lack of *Hand1*-LV lineage cells results in a markedly hypoplastic LV (Fig 2L). At E12.5, the LVs of *Hand1*^*LV*^*-Cre; R26R*^*lacZ/DTA*^ embryos were visibly smaller than *Hand1*^*LV*^*-Cre; R26R*^*lacZ/+*^ controls (Fig 2M and 2N) and showed few X-gal-stained cells when compared to littermates that do not carry the *DTA* allele. Interestingly, these mid-gestation LVs, from which the *Hand1*-lineage has been ablated, do not display perturbed expression of the LV markers *Nppa* and *Gja5* (S2 Fig). Thus, ablation of *Hand1*-lineage LV myocardium between E8.5, when the *Hand1*^*LV*^ enhancer is first upregulated, and E12.5, when the *Hand1*^*LV*^ enhancer begins downregulation, results in a hypoplastic LV.

To follow the biological impact of *Hand1*-LV lineage ablation, we assayed the phenotypes of *Hand1*^*LV*^*-Cre; R26R*^*lacZ/DTA*^ and control embryos at E16.5. To our surprise, *Hand1*^*LV*^*-Cre; R26R*^*lacZ/DTA*^ hearts were indistinguishable in size from controls that lacked the *DTA* allele (Fig 2O and 2P; arrows). X-gal staining confirmed that embryos that contain the *DTA* allele exhibit minimal *Hand1*-lineage cells within their LVs. Although adult cardiomyocytes are refractory to reentering the cell cycle [1, 11–13], embryonic and neonatal cardiomyocytes retain proliferative potential [14–16]. To confirm that the restoration of LV size was simply due to an enhanced cardiomyocyte proliferation from non-*Hand1*-lineage LV myocardium, phospho-Histone-H3 immunostaining was carried out at E12.5 and E14.5 (Fig 2Q–2U). Increased proliferation was observed within the LVs of *Hand1*^*LV*^*-Cre; R26R*^*lacZ/DTA*^ hearts, compared to controls, at E14.5, but not at E12.5 (Fig 2U). *Hand1*^*LV*^*-Cre; R26R*^*lacZ/DTA*^ mice were born at Mendelian ratios (S3A Fig) and were viable and fertile. These hearts lacked gross structural cardiac abnormalities, exhibiting only a subtle rounding of the free LV wall (S3B and S3C Fig). These hearts exhibited cardiac function, as assayed by ejection fraction (EF), fractional shortening (FS), and other echocardiographic parameters, that was indistinguishable from *Cre*-negative controls (S3D–S3O Fig), and within the normal range for adult mice under isoflurane anesthesia [17]. Taken together, these data demonstrate that embryonic ablation of the *Hand1*-lineage is not sufficient to permanently disrupt LV development.

### Myocardial deletion of *Hand1* and *Hand2* within the *Hand1*^*LV*^*-Cre* lineage results in proliferative, morphological, and molecular abnormalities that result in a hypoplastic LV lumen

**Fig 3:**
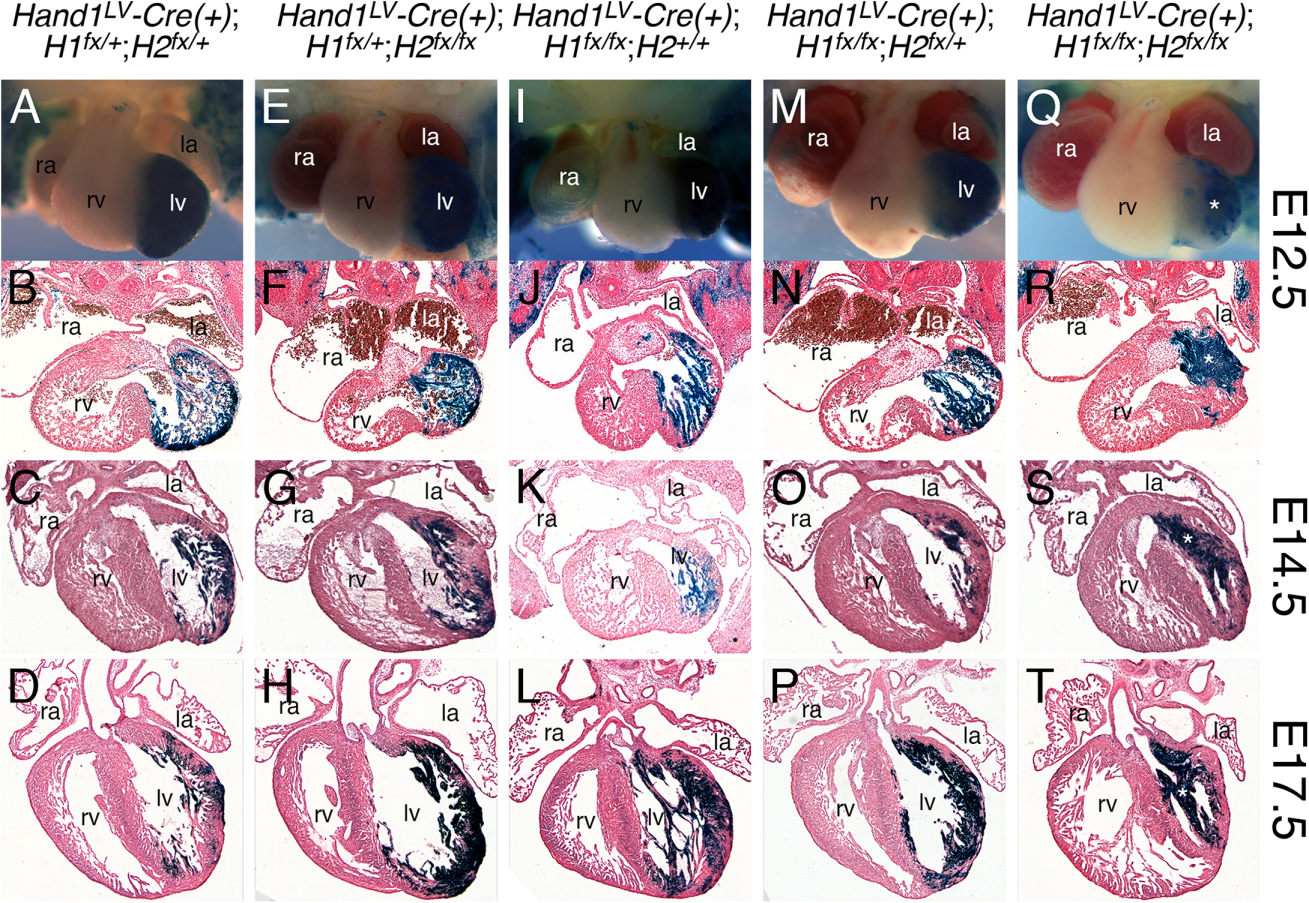
*Hand1*^*LV*^*-Cre*-mediated ablation of *Hand1* and *Hand2* results in a dysmorphic LV, and a single ventricle phenotype perinatally. A-T) *R26R*^*lacZ*^ reporter staining reveals that ablation of both *Hand1* and *Hand2* results in an abnormally thickened ventricular wall (R-T, white asterisks) that reduces the LV chamber lumen at E12.5, E14.5, and E17.5 (n = 4, 4, and 8, respectively).

As we have established that the *Hand1*-LV myocardial lineage is not required for cardiogenesis, we next investigated whether the expression of *Hand1* and *Hand2* within the embryonic LV is required for normal heart development. Although early embryonic expression analysis shows that the majority of *Hand2* expression within the heart is restricted to the endocardium, epicardium, and SHF derived myocardium [10, 18, 19], at later embryonic stages, *Hand2* mRNA becomes detectable within E11.5 LV myocardium in a pattern overlapping with *Hand1* (S4C and S4D Fig). We subsequently generated compound heterozygous *Hand1*^*LV*^*-Cre;Hand1*^*fx/+*^*;Hand2*^*fx/+*^ male mice and crossed them to *Hand1*^*fx/fx*^*;Hand2*^*fx/fx*^ females to generate *Hand1*^*LV*^*-Cre;Hand1*^*fx/fx*^*;Hand2*^*fx/fx*^ offspring (Fig 3). *Hand1*^*LV*^*-Cre;Hand1*^*fx/+*^*;Hand2*^*fx/+*^ (Fig 3A–3D) embryos undergo normal cardiac development and are indistinguishable from *wild type* controls. Similarly, embryos that delete *Hand2* from the LV but retain a single copy of *Hand1* (*Hand1*^*LV*^*-Cre;Hand1*^*fx/+*^*;Hand2*^*fx/fx*^) also exhibit largely normal cardiac development (Fig 3E–3H). Interestingly, consistent with previous studies [9], deletion of *Hand1* from the LV (*Hand1*^*LV*^*-Cre;Hand1*^*fx/fx*^) results in a morphologically disorganized LV, wherein *Hand1*-lineage cells appear to localize more to trabeculations compared to the compact zone (Fig 3I–3L). At E17.5, abnormal cardiomyocytes localize within the LV lumen (Fig 3L) and ventricular septal defects are also observed (S5H Fig). Deletion of *Hand1* from the LV leaving a single copy of *Hand2* (*Hand1*^*LV*^*-Cre;Hand1*^*fx/fx*^*;Hand2*^*fx/+*^) results in a similar phenotype (Fig 3M–3P, S5J–S5L Fig). Complete deletion of Hand factors from the LV (*Hand1*^*LV*^*-Cre;Hand1*^*fx/fx*^*;Hand2*^*fx/fx*^) caused a pronounced internalization of the *Hand1*-lineage cells where X-gal staining in whole mount is noticeably opaque and in section reveals *Hand1*-lineage cells occluding the LV lumen (Fig 3Q–3S). Histological analysis at E17.5 revealed severely occluded LV lumen and poorly formed IVS (Fig 3T, S5M–S5O Fig), as well as a double outlet right ventricle (black arrowheads, S5N Fig), and hyperplastic mitral valves (S5O Fig), although the aorta and OFT valves are phenotypically indistinguishable from controls (S5M Fig). These phenotypes are summarized in Table 1. These data suggest that a Hand factor loss-of-function within the LV myocardium alters chamber morphology via a *Hand* gene dosage dependent mechanism, in which *Hand1*-lineage cells are found less frequently in the compact zone at the expense of increased cells within the LV lumen representing trabecular and papillary muscle cardiomyocytes.

**Table 1:** Cardiac defects in *Hand1*;*Hand2* conditional knockouts at E17.5.

Genotype	n	VSD	hyperplastic mitral valves	abnormal LV trabeculae	overgrown LV myocardium	Phenotypically Normal
*H1*^*fx/fx*^;*H2*^*fx/fx*^;*(-)*	2	0 (0%)	0 (0%)	0 (0%)	0 (0%)	2 (100%)
*H1*^*+/fx*^;*H2*^*+/fx*^;*Cre(+)*	6	0 (0%)	0 (0%)	0 (0%)	0 (0%)	6 (100%)
*H1*^*+/fx*^;*H2*^*fx/fx*^;*Cre(+)*	4	0 (0%)	0 (0%)	0 (0%)	0 (0%)	4 (100%)
*H1*^*fx/fx*^;*H2*^*+/+*^;*Cre(+)*	4	2 (50%)	0 (0%)	1 (25%)	0 (0%)	2 (50%)
*H1*^*fx/fx*^;*H2*^*+/fx*^;*Cre(+)*	5	1 (20%)	3 (60%)	3 (60%)	0 (0%)	1 (20%)
*H1*^*fx/fx*^;*H2*^*fx/fx*^;*Cre(+)*	8	6 (75%)	7 (87.5%)	3 (37.5%)	5 (62.5%)	0 (0%)

VSD–ventricular septal defect.

### Surviving myocardial *Hand1*^*LV*^*-Cre;Hand1*^*fx/fx*^*;Hand2*^*fx/fx*^ exhibit compromised cardiac function

**Fig 4:**
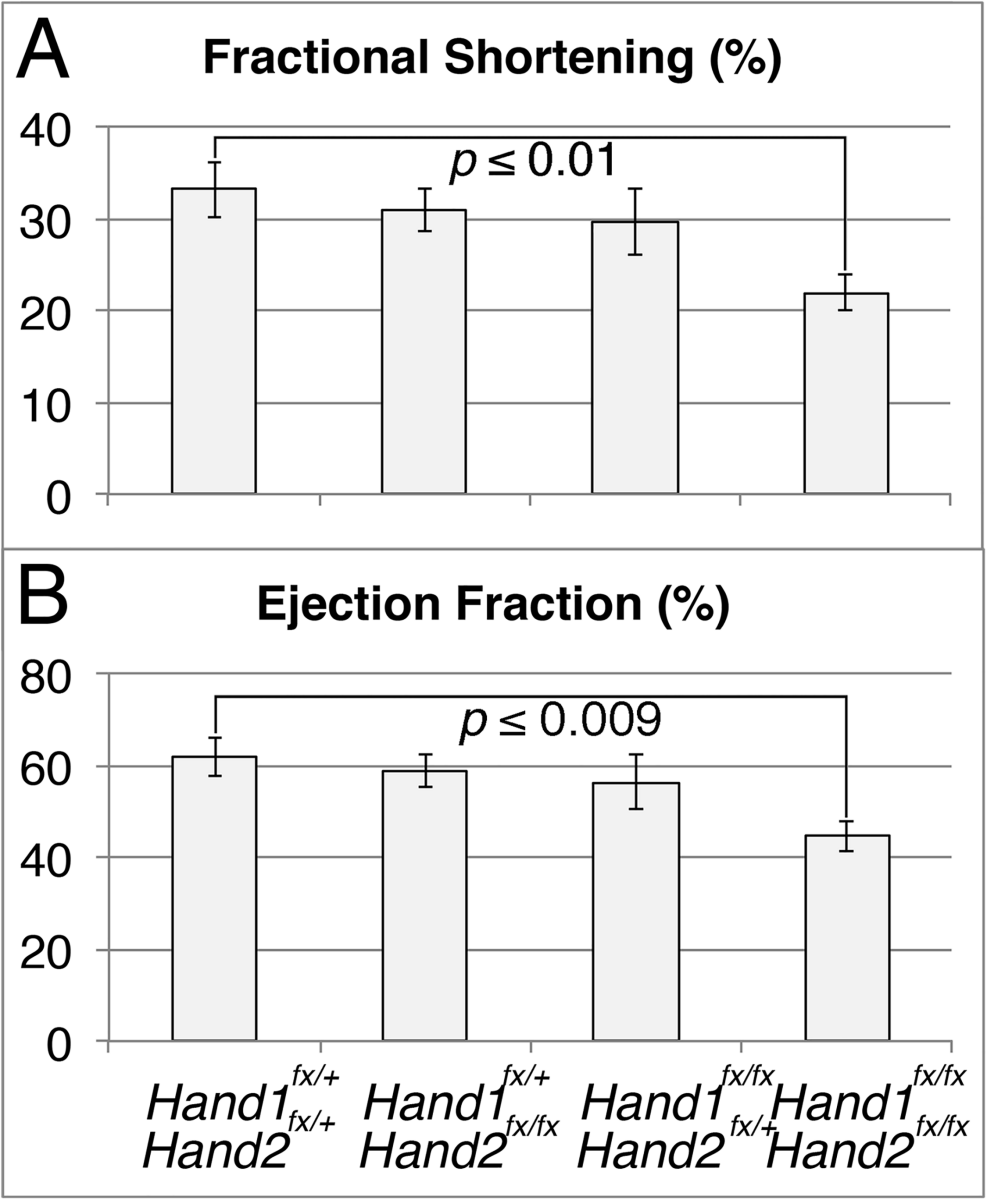
*Hand1*^*LV*^*-Cre*-mediated *Hand1*;*Hand2* CKOs have impaired cardiac function as adults. Mice at P56 reveal significant decreases in fractional shortening (A) and ejection fraction (B) in *Hand1*^*LV*^*-Cre*;*Hand1*^*fx/fx*^*;Hand2*^*fx/fx*^ mutants (n = 8) relative to *Hand1*^*LV*^*-Cre*;*Hand1*^*fx/+*^*;Hand2*^*fx/+*^ littermates (n = 8). Data are represented as mean ± standard error of mean.

Despite the morphological defects observed in *Hand1*^*LV*^*-Cre;Hand1*^*fx/fx*^*;Hand2*^*fx/fx*^ embryos, *Hand* loss-of-function mice survive perinatally at approximately 50% of the expected Mendelian ratio (Table 2). Indeed, although *Hand1*^*LV*^*-Cre;Hand1*^*fx/fx*^*;Hand2*^*fx/fx*^ pups are underrepresented, this underrepresentation is not statistically significant (Table 2). Echocardiographic analysis of P56 *Hand1*^*LV*^*-Cre;Hand1*^*fx/fx*^*;Hand2*^*fx/fx*^ survivors (S6 Fig) revealed that systolic function (fractional shortening and ejection fraction) is significantly compromised in *Hand1LV-Cre;Hand1*^*fx/fx*^*;Hand2*^*fx/fx*^ mice (Fig 4), whereas other measures of LV morphology and function, such as chamber dimensions and wall thickness, were not significantly altered (S7 Fig).

**Table 2:** Expected and actual survival data of intercrosses of *Hand1*^*LV*^*-Cre; Hand1*^*fx/+*^*;Hand2*^*fx/+*^ and *Hand1*^*fx/fx*^;*Hand2*^*fx/fx*^ mice.

Genotype	Expected (n = 123)	Recovered
*H1*^*+/fx*^;*H2*^*+/fx*^;*(-)*	15.4	13 (1)
*H1*^*+/fx*^;*H2*^*fx/fx*^;*(-)*	15.4	20
*H1*^*fx/fx*^;*H2*^*+/fx*^;*(-)*	15.4	12
*H1*^*fx/fx*^;*H2*^*fx/fx*^;*(-)*	15.4	14
*H1*^*+/fx*^;*H2*^*+/fx*^;*Cre(+)*	15.4	25
*H1*^*+/fx*^;*H2*^*fx/fx*^;*Cre(+)*	15.4	13
*H1*^*fx/fx*^;*H2*^*+/fx*^;*Cre(+)*	15.4	17
*H1*^*fx/fx*^;*H2*^*fx/fx*^;*Cre(+)*	15.4	9 (1)

Numbers in parentheses denote pups that died prior to weaning.

Chi squared equals 11.829 with 7 degrees of freedom.

The two-tailed P value equals 0.1063

### LV cardiomyocytes are mis-specified in *Hand1*^*LV*^*-Cre;Hand1*^*fx/fx*^*;Hand2*^*fx/fx*^ mice

**Fig 5:**
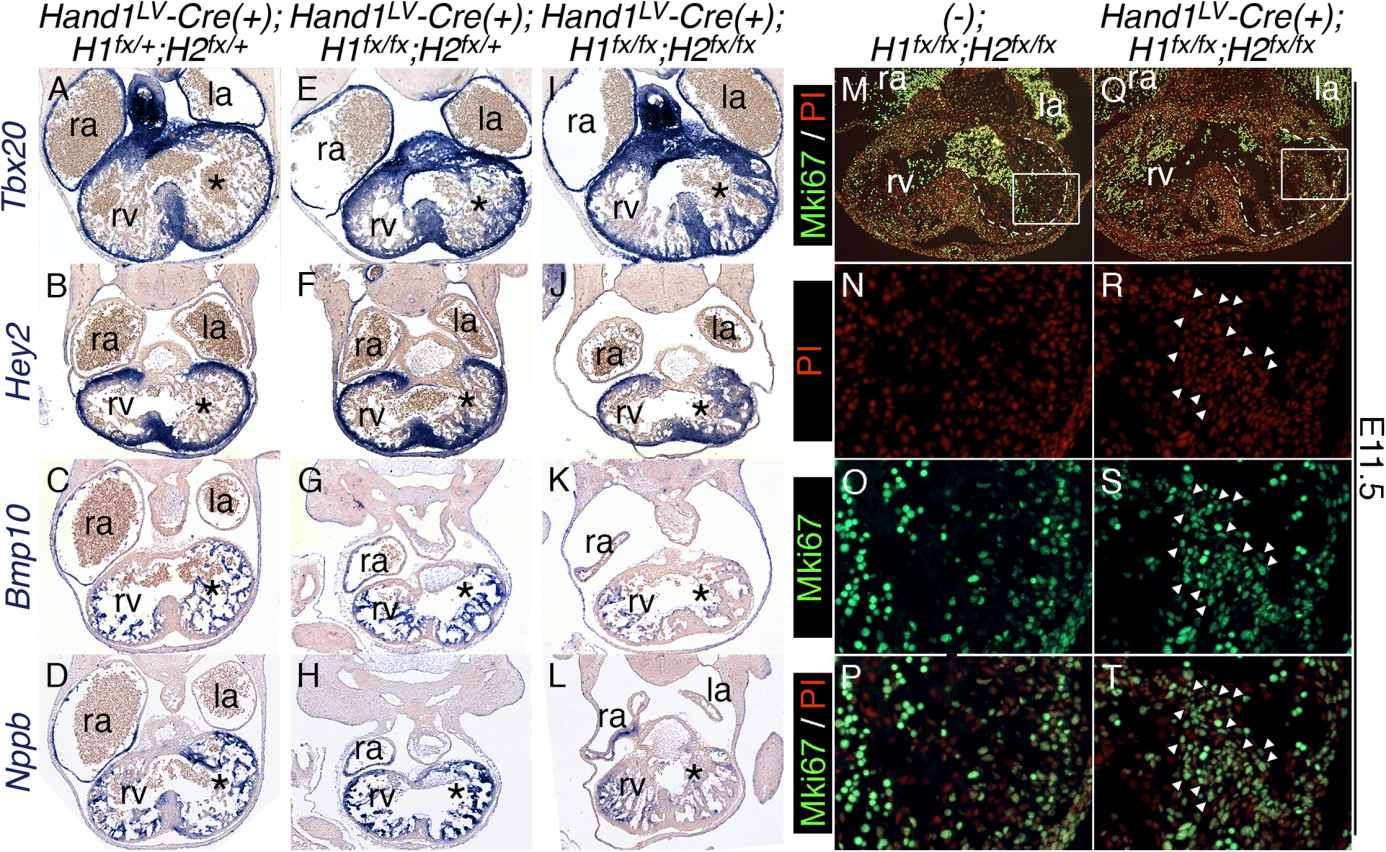
*Hand1*^*LV*^*-Cre*-mediated *Hand1*;*Hand2* CKOs display abnormal myocardial gene expression and trabecular proliferation in the LV. A, B, E, F, I, J) Section *in situ* hybridization of E11.5 hearts showing that the transcription factors *Tbx20* and *Hey2* are both expressed throughout both the presumptive compact myocardium and IVS, but excluded from the trabecular myocardium in control hearts (A, B). This sharp delineation between compact and trabecular myocardium is lost in the LVs of *Hand1*^*LV*^*-Cre*;*Hand1*^*fx/fx*^*;Hand2*^*fx/+*^ hearts (E, F). In *Hand1*^*LV*^*-Cre*;*Hand1*^*fx/fx*^*;Hand2*^*fx/fx*^ hearts, most of the trabeculae in the LV ectopically express compact myocardium markers, whereas the RV trabeculae do not (I, J; n = 4). C, D, G, H, K, L) Section *in situ* hybridization of E11.5 hearts showing that *Bmp10* and *Nppb* are both expressed throughout the trabeculae, and are largely excluded from the compact myocardium in control hearts (C, D). In *Hand1*^*LV*^*-Cre*;*Hand1*^*fx/fx*^*;Hand2*^*fx/fx*^ hearts, *Bmp10* and *Nppb* expression is downregulated within the trabeculae (K, L; n = 4). M-T) Immunohistochemistry for Mki67 on E11.5 *Control* (M-P) and *Hand1*^*LV*^*-Cre*;*Hand1*^*fx/fx*^*;Hand2*^*fx/fx*^ hearts (Q-T). Dashed lines delimit the boundary of trabeculae and compact zone. Boxes in M and Q denote high magnification images in N-P and R-T, respectively. White arrowheads in R, S and T highlight Mki67-positive nuclei. la–left atrium, ra–right atrium, rv–right ventricle, PI–propidium iodide. Asterisks denote the left ventricle.

We next sought to characterize the etiology of the luminal cardiomyocyte overgrowth in *Hand1*^*LV*^*-Cre;Hand1*^*fx/fx*^*;Hand2*^*fx/fx*^ embryos. To this end, we performed marker analyses to characterize distinct subpopulations of cardiomyocytes in the heart. The following expression studies revealed no appreciable difference between *(-);Hand1*^*fx/fx*^*;Hand2*^*fx/fx*^, *Hand1*^*LV*^*-Cre(+)*; *Hand1*^*fx/+*^*;Hand2*^*fx/+*^, and *Hand1*^*LV*^*-Cre(+)*;*Hand1*^*fx/+*^*;Hand2*^*fx/ fx*^ embryos. For ease of presentation, *Hand1*^*LV*^*-Cre(+)*; *Hand1*^*fx/+*^*;Hand2*^*fx/+*^ embryos are presented as controls. *Tbx20* and *Hey2* expression marks compact and IVS myocardium [20, 21]. Section *in situ* hybridization of E11.5 hearts showed that *Tbx20* and *Hey2* are both expressed throughout the presumptive compact myocardium and excluded from the trabecular myocardium in control hearts (Fig 5A and 5B). This sharp delineation between compact and trabecular myocardium is lost in the LVs of *Hand1*^*LV*^*-Cre(+)*; *Hand1*^*fx/fx*^*;Hand2*^*fx/+*^ hearts (Fig 5E and 5F; asterisks). In *Hand1*^*LV*^*-Cre;Hand1*^*fx/fx*^*;Hand2*^*fx/fx*^ hearts, most of the trabeculae within the LV ectopically express compact myocardium markers, whereas the RV trabeculae do not (Fig 5I and 5J). Conversely, the trabecular markers *Bmp10* and *Nppb* show robust expression throughout the trabeculae, and expression is largely excluded from the compact myocardium in control hearts (Fig 5C and 5D). In *Hand1*^*LV*^*-Cre;Hand1*^*fx/fx*^*;Hand2*^*fx/fx*^ hearts, *Bmp10* and *Nppb* expression is downregulated within the LV trabeculae when compared to RV trabeculae (Fig 5K and 5L; asterisks). We conclude that *Hand1*^*LV*^*-Cre;Hand1*^*fx/fx*^*;Hand2*^*fx/fx*^ hearts display ectopic compact myocardium marker expression and reduced trabecular marker expression within the LV.

### *Hand1*^*LV*^*-Cre;Hand1*^*fx/fx*^*;Hand2*^*fx/fx*^ hearts display abnormal trabecular proliferation in the LV

By Carnegie Stage 16 in the human embryo (equivalent to E11.5 in mice) proliferation in the ventricular trabeculae has declined [22]. We next sought to correlate differences in the proliferative capacity of compact and trabecular myocardium with the overgrowth seen in *Hand1*^*LV*^*-Cre;Hand1*^*fx/fx*^*;Hand2*^*fx/fx*^ LVs. Mki67 marks cells actively undergoing cell cycle, but is excluded from cells in G_0_ [23]. Mki67 immunohistochemistry revealed that, in control embryos, nuclear Mki67 is robustly detected within compact myocardium and the IVS, but is largely excluded from trabecular myocardium (Fig 5M–5P). In contrast, *Hand1*^*LV*^*-Cre;Hand1*^*fx/fx*^*;Hand2*^*fx/fx*^ trabecular cardiomyocytes show robust Mki67 staining (Fig 5Q–5T; arrowheads). These findings indicate that cardiomyocyte proliferation is dysregulated within the LV of *Hand1*^*LV*^*-Cre;Hand1*^*fx/fx*^*;Hand2*^*fx/fx*^ embryos.

### *Hand1*^*LV*^*-Cre;Hand1*^*fx/fx*^*;Hand2*^*fx/fx*^ hearts display abnormal expansion of ventricular septum markers into the LV

**Fig 6:**
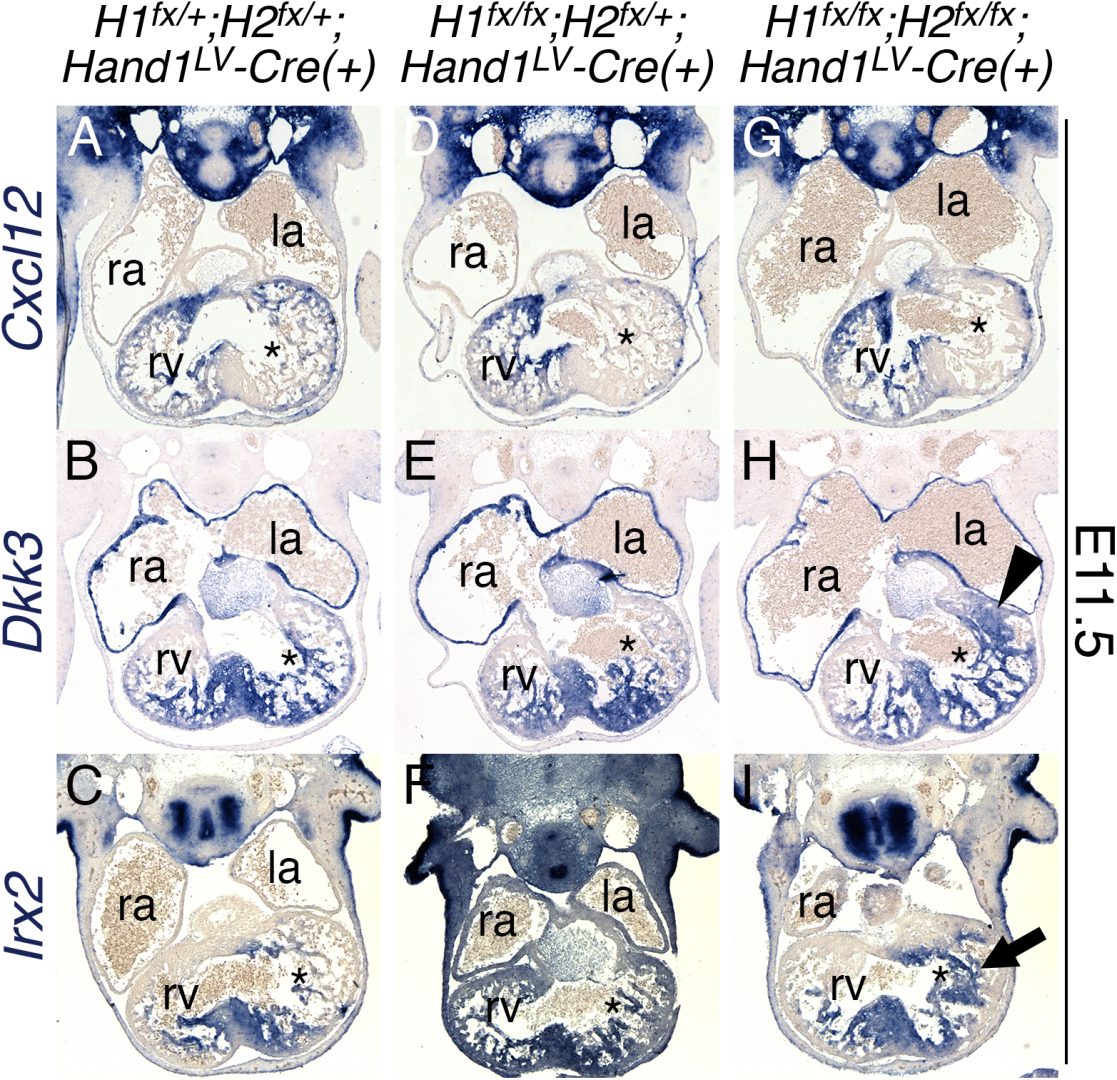
*Hand1*^*LV*^*-Cre*-mediated *Hand1*;*Hand2* CKOs display abnormal expansion of ventricular septum markers into the LV. A-I) Section *in situ* hybridization of E11.5 hearts showing expression of the chemokine *Cxcl12* (A, D, G), the secreted Wnt inhibitor *Dkk3* (B, E, H), and the transcription factor *Irx2* (C, F, I) in control (A-C), *Hand1* heterozygous; *Hand2* CKO (D-F), and *Hand1*^*LV*^*-Cre*;*Hand1*^*fx/fx*^*;Hand2*^*fx/fx*^ hearts (G-I; n = 4). Black arrowhead (H) indicates the expansion of the *Dkk3* expression domain. Black arrow (I) indicates the expansion of the *Irx2* expression domain. la–left atrium, ra–right atrium, rv–right ventricle. Asterisks denote the left ventricle.

Previous studies have reported that ectopic *HAND1* expression throughout the heart disrupts IVS formation [24]. We reasoned that the abnormal proliferation and marker expression seen in *Hand1*^*LV*^*-Cre;Hand1*^*fx/fx*^*;Hand2*^*fx/fx*^ hearts may reflect aberrant ventricular septogenesis. Section *in situ* hybridization of E11.5 hearts showed that expression of the chemokine *Cxcl12* is strong in the free walls of the ventricles, but is largely excluded from the IVS (Fig 6A). In both *Hand1*^*LV*^*-Cre;Hand1*^*fx/fx*^*;Hand2*^*fx/+*^. and *Hand1*^*LV*^*-Cre;Hand1*^*fx/fx*^*;Hand2*^*fx/fx*^ hearts, LV *Cxcl12* expression is excluded specifically from the trabecular myocardium, in addition to the left side of the ventricular septum (Fig 6D and 6G). The secreted Wnt inhibitor *Dkk3* and the transcription factor *Irx2* are both markers of the IVS [25]. Expression of both *Dkk3* (Fig 6H; black arrowhead) and *Irx2* (Fig 6I; black arrow) expands toward the atrioventricular canal in *Hand1*^*LV*^*-Cre;Hand1*^*fx/fx*^*;Hand2*^*fx/fx*^ hearts. Together, these data indicate that a loss of both Hand1 and Hand2 function within the LV causes an expansion of the IVS gene expression program.

### DTA ablation rescues the lethality and cardiac dysfunction seen in *Hand1;Hand2* conditional knockouts

**Fig 7:**
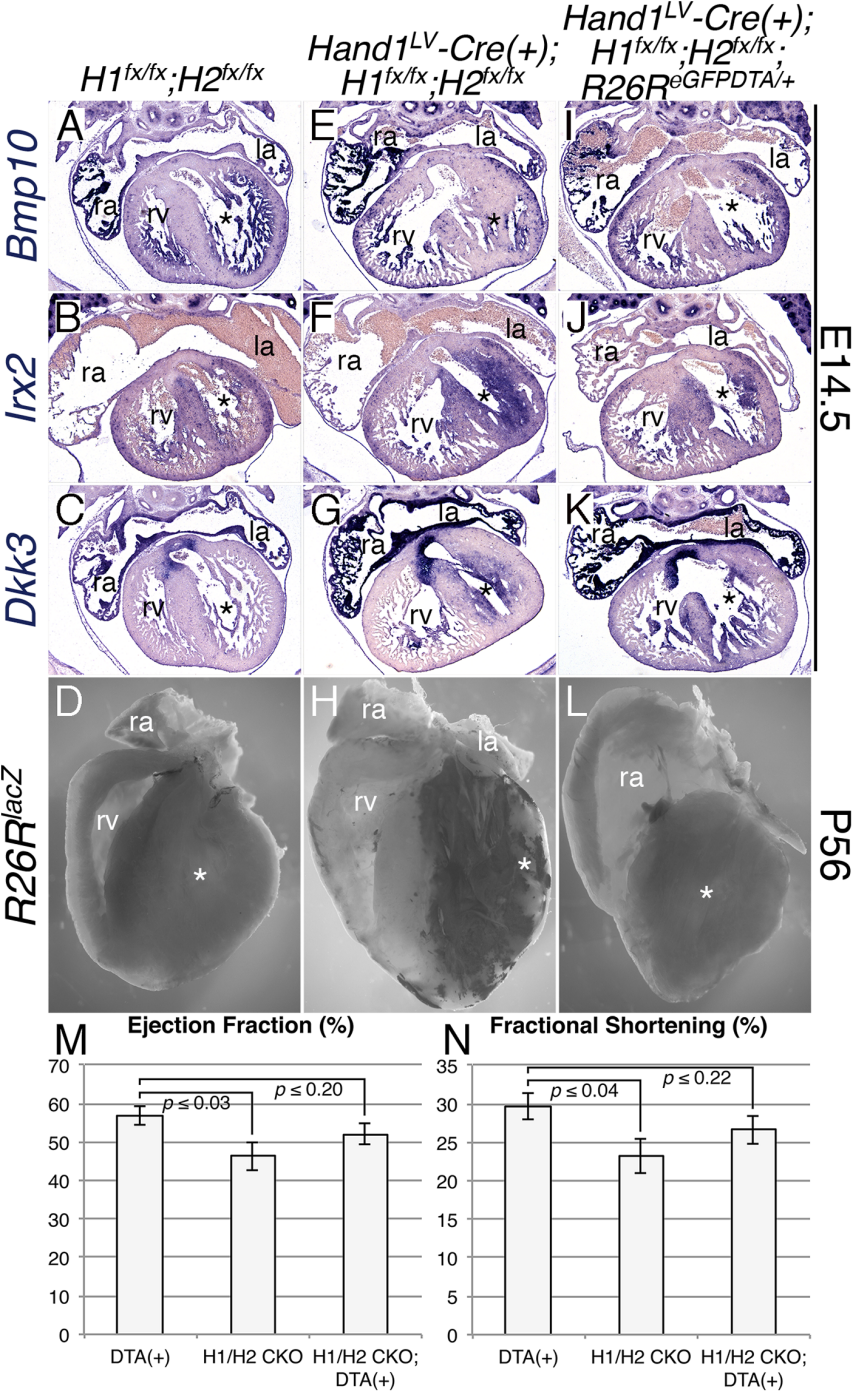
DTA ablation rescues the lethality and cardiac dysfunction seen in *Hand1*^*LV*^*-Cre Hand1*;*Hand2* CKOs. A-K) Section *in situ* hybridization showing expression of *Bmp10* (A, E, I), *Irx2* (B, F, J), *Dkk3* (C, G, K) in E14.5 embryonic hearts, and X-gal-stained bisected P56 *Control* (D), *Hand1*^*LV*^*-Cre*;*Hand1*^*fx/fx*^*;Hand2*^*fx/fx*^ (H), and *Hand1*^*LV*^*-Cre*;*Hand1*^*fx/fx*^*;Hand2*^*fx/fx*^;*R26R*^*DTA*^-rescued hearts (L). Note that the RV of the heart in panel L has been mechanically damaged. (M, N) Echocardiographic analyses of P56 mice revealed restoration of ejection fraction (M) and fractional shortening (N) in *Hand1*^*LV*^*-Cre(+);Hand1*^*fx/fx*^*;Hand2*^*fx/fx*^*;R26R*^*DTA*^ mice (n = 15) compared to *Hand1*^*LV*^*-Cre(+);Hand1*^*fx/fx*^*;Hand2*^*fx/fx*^ CKOs. *Hand1*^*LV*^*-Cre(-);R26R*^*DTA*^ mice, denoted as DTA(+), are included as controls. Data are represented as mean ± standard error of mean.

Given that the embryonic heart can recover from DTA-mediated ablation of *Hand1*-lineage cardiomyocytes, we tested whether ablation of *Hand1*;*Hand2*-null cardiomyocytes can rescue the phenotypes associated with their loss-of-function. The *R26R*^*DTA*^ allele was bred onto a *Hand1*^*LV*^*-Cre*;*Hand1*^*fx/fx*^*;Hand2*^*fx/fx*^ background. E14.5 section *in situ* hybridization of control hearts (Fig 7A–7C) showed expected expression patterns for *Bmp10*, *Irx2*, and *Dkk3* within the trabeculae and IVS respectively. As expected *Hand1*^*LV*^*-Cre*;*Hand1*^*fx/fx*^*;Hand2*^*fx/fx*^ hearts showed reduced *Bmp10* LV expression along with expanded *Dkk3*- and *Irx2* LV cardiomyocyte expression (Fig 7E–7G). These changes in gene expression are reversed in *Hand1*^*fx/fx*^*;Hand2*^*fx/fx*^*;Hand1*^*LV*^*-Cre(+);R26R*^*DTA*^ embryos (Fig 7I–7K). Morphological examination of X-gal-stained bisected P56 hearts revealed heart morphology indistinguishable from controls (Fig 7D, S8A Fig), in contrast to the overgrowth of *Hand1*-lineage cardiomyocytes that occlude the lumen of *Hand1*^*LV*^*-Cre*;*Hand1*^*fx/fx*^*;Hand2*^*fx/fx*^ hearts (Fig 7H, X-gal stained myocardium; S8B Fig). Occluded LV lumens are absent in *Hand1*^*LV*^*-Cre*;*Hand1*^*fx/fx*^*;Hand2*^*fx/fx*^*;R26R*^*DTA*^ embryos (Fig 7L, S8C and S8D Fig). Lineage tracing reveals that the majority of lacZ-positive cells are ablated in these hearts; however, consistent with DTA-ablation embryos (Fig 2P), small populations of lacZ-positive cells were sometimes detectable within these hearts (S8D Fig), which we interpret as cells that have recombined only the *lacZ* reporter, but not the *DTA* allele. *Hand1*^*LV*^*-Cre*;*Hand1*^*fx/fx*^*;Hand2*^*fx/fx*^*;R26R*^*DTA*^ pups survive at Mendelian ratios (Table 3) and display restored ejection fraction (Fig 7M) and fractional shortening (Fig 7N). Again, other measures of cardiac function were not significantly altered (S8E–S8J Fig).Together, these data demonstrate that ablation of mutant cardiomyocytes from the developing LV restores cardiac function.

**Table 3:** Expected and actual survival data of intercrosses of *Hand1*^*LV*^*-Cre; Hand1*^*fx/fx*^*;Hand2*^*fx/fx*^ and *Hand1*^*fx/fx*^;*Hand2*^*fx/fx*^;*R26R*^*+/eGFPDTA*^ mice.

Genotype	Expected (n = 77)	Recovered
*H1*^*fx/fx*^;*H2*^*fx/fx*^;*(-)*;*+/+*	19.25	19
*H1*^*fx/fx*^;*H2*^*fx/fx*^;*(-)*;*DTA/+*	19.25	31
*H1*^*fx/fx*^;*H2*^*fx/fx*^;*Cre(+)*;*+/+*	19.25	8
*H1*^*fx/fx*^;*H2*^*fx/fx*^;*Cre(+)*;*DTA/+*	19.25	19

Chi squared equals 13.753 with 3 degrees of freedom.

The two-tailed P value equals 0.0033.

## Discussion

CHDs that alter the LV exhibit poor clinical outcomes [5, 26]. The inability to interrogate LV gene expression in isolation has limited the ability to understand the molecular mechanisms that drive LV morphogenesis. This study reports the generation of a novel LV-restricted *Cre* driver line that allows for such focused interrogation. First, we ablated *Hand1*-lineage cardiomyocytes within an E9.0-E13.5 developmental window. As expected, LV size was greatly reduced by E10.5, and this reduced size remains clear at E14.5 (Fig 2). *Hand1* cardiac expression is downregulated by E13.5 [27]. We observed a significant increase in LV cardiomyocyte cell proliferation at E14.5 (Fig 2U). By E16.5, LV size is indistinguishable from control hearts (Fig 2O and 2P). These findings support published data showing that embryonic and up to 7-day postnatal cardiomyocytes retain regenerative potential [14–16], and further demonstrate that the *Hand1*-lineage can be ablated from the developing LV, and the heart can nonetheless undergo full regenerative repair. It is also clear that these replacement cardiomyocytes do not express *Hand1*—if they did, *Cre* would also be expressed, thereby activating *DTA* expression and killing the replacement cell. That said, conclusive identification of the origin(s) of the cells that replace the ablated *Hand1*-lieage cells will require further study employing, for example, dual lineage tracing systems. A candidate for this progenitor population is the SHF. One of the most well studied markers of the SHF is the *Isl1-Cre* [28]. Although it is excluded from the majority of LV cardiomyocytes, *Isl1-Cre*-lineage cells do appear in the LV [28–31], and it is possible that this relatively minor population expands in the absence of *Hand1*-lineage cells. Indeed, this would explain why early stage DTA-ablation embryos, despite almost completely lacking *Cre*-lineage cells, are grossly similar to non-ablated littermates (Fig 2I and 2J). If this were the case, it would indicate that SHF-derived cardiomyocytes are sufficient to replace PHF-derived cardiomyocytes. Regardless of potential contributions from other cardiomyocyte lineages, including the SHF, these observations indicate that *Hand1*-lineage cells are not required for cardiac development, and that, through elevated proliferation, a *Hand1*-negative fated cardiomyocyte population within the developing heart is sufficient to generate a functional LV.

In contrast, Hand1 and Hand2 are required for normal LV morphogenesis. By E11.5, both Hand genes are expressed within the LV myocardium (S4 Fig). Loss of *Hand1*, and, more severely, *Hand1* and *Hand2*, leads to abnormal LV morphology characterized by expanded compact and IVS marker expression (*Tbx20*, *Hey2*, *Dkk3*, *Irx2*; Figs. 5 and 6) and correspondingly reduced trabecular marker expression. An increase in Mki67-positive nuclei (Fig 5M–5T) within the LV trabecular zone indicates that aberrant cell proliferation causes the observed LV hyperplasia. Taking these observations together, we conclude that the expanding cardiomyocyte population observed in *Hand1*^*LV*^*-Cre*;*Hand1*^*fx/fx*^*;Hand2*^*fx/fx*^ mutants is derived from myocardium that has ectopically activated the IVS gene expression program. The *Hand1*-lineage marks very little of the IVS [7]. Proliferation in *Hand1*^*LV*^*-Cre;Hand1*^*fx/fx*^*;Hand2*^*fx/fx*^ mutant hearts is not restricted to this small domain of *Hand1*-expressing IVS cells on the inner wall near the cardiac apex. This suggests that some *Hand1*-lineage compact zone myocardium adopts an IVS cell-like fate. The observation that the *Hand1*-lineage within *Hand1*^*LV*^*-Cre;Hand1*^*fx/fx*^*;Hand2*^*fx/fx*^ mutant hearts is largely excluded from the LV compact zone, and is instead localized to the inner curvature supports this finding. These results are especially interesting in light of recent studies, which propose that LVNC likely results from abnormal growth of compact myocardium [32]. It would be informative to test whether IVS markers also expand into luminal domains in such models.

In addition to LVNC, these *Hand1*^*LV*^*-Cre;Hand1*^*fx/fx*^*;Hand2*^*fx/fx*^ mutants share certain phenotypic similarities with HLHS patients; however, they lack the aortic valve phenotypes characteristic of HLHS (S5M Fig). A recently published mouse model of HLHS [33] posits a digenic etiology of HLHS, in which dysfunction of one gene disrupts cardiomyocyte proliferation and differentiation to cause LV hypoplasia, while a second mutation causes aortic valve abnormalities. Importantly, these findings provide evidence that the aortic phenotypes are not secondary to the LV phenotypes, and therefore, the lack of *Hand1*^*LV*^*-Cre;Hand1*^*fx/fx*^*;Hand2*^*fx/fx*^ aortic defects is not surprising. As mentioned, a subset of HLHS patients displays *HAND1* mutations [8]. *Hand1* is expressed in the OFT [27] as well as the LV, and as such could have pleiotropic functions in both aortic valve and LV development; however, ablation of *Hand1* in the neural crest progenitors of the aortic valves is not pathogenic [34]. Given that this study reports the unexpected finding that Hand1 and Hand2 have overlapping roles in LV development, it would be of interest to reevaluate Hand1 function in the OFT considering potential functional redundancy with Hand2.

Increased cardiomyocyte proliferation can negatively impact specification [14, 15]. The *Hand1*^*LV*^*-Cre*;*Hand1*^*fx/fx*^*;Hand2*^*fx/fx*^ LV hyperplasia occludes so much of the LV lumen that it results, in severe cases, in a single ventricle phenotype (Fig 3T, S5N Fig). In spite of the significant LV hyperplasia, 50% of *Hand1*^*LV*^*-Cre*;*Hand1*^*fx/fx*^*;Hand2*^*fx/fx*^ mutants survive to birth and live to adulthood. These surviving individuals exhibit compromised systolic function (Fig 4). It is clear that both Hand1 and Hand2 contribute to this phenotype, and that Hand1 plays a more significant role. *Hand1*^*LV*^*-Cre*;*Hand1*^*fx/+*^*;Hand2*^*fx/fx*^ mutants display no observable cardiac phenotypes, whereas *Hand1*^*LV*^*-Cre*;*Hand1*^*fx/fx*^*;Hand2*^*+/+*^ and *Hand1*^*LV*^*-Cre*;*Hand1*^*fx/x*^*;Hand2*^*fx/+*^ mutants show both morphological and molecular changes in gene expression. Given that *Hand2* LV expression is not detectable until E11.5, and its LV expression is not dependent upon Hand1, *Hand1* mutants are less severe likely due to the later expression of Hand2.

Finally, we observe a functional rescue of *Hand1*^*LV*^*-Cre*;*Hand1*^*fx/fx*^*;Hand2*^*fx/fx*^ mutants when mutant cells are ablated via co-activation of DTA expression (Fig 7). Given that heart development does not require the *Hand1*-lineage (Fig 2) this may not be surprising. Nevertheless, this observation suggests that removal of molecularly abnormal cardiomyocytes from a developing heart could be beneficial if molecularly normal cells are present and are still proliferative.

## Materials and methods

All relevant data are within the manuscript and its Supporting Information files.

### Transgenic mice

The Indiana University Transgenic and Knock-Out Mouse Core generated the *Hand1*^*LV*^*-Cre* transgenic mouse line on a C3HeB/FeJ background. Genotyping of the *Hand1*^*tm2Eno*^, *Hand2*^*tm1Cse*^, *Gt(ROSA)26Sor*^*tm1(DTA)Jpmb*^, and *Gt(ROSA)26Sor*^*tm1Sor*^ alleles has previously been described [9, 10, 35, 36]. These mice were maintained on a mixed C57Bl/6;129S background. Embryos were not selected for sex, and were evaluated blindly for all analyses. Mice and other reagents are available from the authors upon request.

### Cloning

To generate *the Hand1*^*LV*^*-Cre* transgene, the genomic sequence corresponding to chr11:57660605–57661348 (in the mm9 assembly​) was cloned 5’ to a modified *eGFP-Cre* vector driven by the basal *Hand1* 2.7kb promoter. Mice were genotyped for the *Cre* allele either via Southern blot or PCR using the primers Cre(F) 5’-CGTACTGACGGTGGGAGAAT-3’ and Cre(R) 5’-TGCATGATCTCCGGTATTGA-3’, with the internal controls Smad4(F) 5’-TAAGAGCCACAGGGTCAAGC-3’ and Smad4(R) 5’-TTCCAGGAAAAACAGGGCTA-3’.

### X-gal staining and histology

X-gal staining was performed as previously described [7, 19, 37, 38].

### Lysotracker and TUNEL

Cell death analysis on control and mutant embryos was performed as described [39]. *Lysotracker* (Life Technologies) was incubated with embryos as per the manufacturer's instructions. Embryos were imaged in a well slide on a Leica DM5000 B compound florescent microscope. TUNEL analyses were performed upon sectioned embryos using the *ApopTag Plus* Fluorescein *in situ* Apoptosis detection kit (S7111 Chemicon International) as per manufacturer’s instructions. TUNEL-positive cells occupying the free wall of the LV and RV (including the myocardial outflow tract) were counted every other section. Significance was determined by student’s t-test.

### Immunohistochemistry and quantification of cell proliferation

Immunohistochemistry was performed as previously described [19, 40–42]. β-galactosidase (Aves BGL-1010; 1:200 dilution), Mlc2v (Synaptic Systems 310 111; 1:500), PECAM (BD Pharmingen 550274; 1:200), Phospho-Histone H3 (Abcam 4797; 1:500), and Mki67 (Dako; 1:500) antibodies were used with biotinylated secondary antibodies and streptavidin-conjugated DyLight 488 or 594 fluorophores (ThermoFisher). Images were collected on a Leica DM5000 B microscope and Leica Application Suite software.

Cell proliferation was assayed via counting of phospho-histone H3 positive nuclei. Left and right ventricles from E12.5 and E14.5 immunostained images were manually isolated in Adobe Photoshop, and nuclei were manually counted using Image J software. For E12.5, n≥9 sections, and for E14.5, n≥10 sections per heart were counted. Total samples analyzed were as follows: E12.5, control embryos (*Hand1*^*LV*^*-Cre(-); R26R*^*lacZ/DTA*^)–n = 2, RV, 35057 cells, total, LV, 35250 cells, total; LV-ablated embryos (*Hand1*^*LV*^*-Cre(+); R26R*^*lacZ/DTA*^)–n = 5, RV, 81471 cells, total, LV, 67618 cells, total; E14.5, control embryos (*Hand1*^*LV*^*-Cre(-); R26R*^*lacZ/DTA*^ or *Hand1*^*LV*^*-Cre(+); R26R*^*lacZ/+*^)–n = 3, RV, 76254 cells, total, LV, 85121 cells, total; LV-ablated embryos–n = 4, RV, 167897 cells, total, LV, 80894 cells, total.

### Echocardiography

Mice were lightly anesthetized with mixture of 1% to 1.5% isoflurane and 100% oxygen while supine on a heated platform. The heart rate were stabilized at 400 to 500 beats per minute before image recording. Images were obtained with a high resolution Micro-Ultrasound system (Vevo 2100, VisualSonics Inc, Toronto, Canada) equipped with a 40-MHz mechanical scan probe. Two-dimensional images were recorded in the parasternal long and short-axis to guide M-mode recordings in the mid-ventricular level. LV systolic function was computed from M-mode measurement according to the recommendations of American Society of Echocardiography committee [43].

### Ethics statement

Animal work was approved by the Indiana University School of Medicine Animal Care and use committee (IACUC) via protocol 10809 Issued to ABF.

## Supporting information

S1 Fig *Hand1*^*LV*^*-Cre; R26R*^*lacZ*^–lineage cells localize primarily to the myocardium, not the endocardium. A-H) Immunohistochemistry on E15.5 *Hand1*^*LV*^*-Cre(+)*;*R26R*^*+/lacZ*^ hearts for β-galactosidase (C, D), Mlc2v (E), and PECAM (F) show that the *Hand1*^*LV*^*-Cre* lineage is predominantly myocardial (yellow co-localization in G), and not endocardial (H). The apical free wall of the LV is shown. Sections are counterstained with DAPI (A, B).
(TIFF)

S2 Fig *Hand1*^*LV*^*-Cre; R26R*^*DTA*^ embryos do not exhibit abnormal LV marker gene expression. A-D) Section *in situ* hybridization of E11.5 hearts showing expression of LV markers *Nppa* (A, B) and *Gja5* (C, D). la–left atrium, lv–left ventricle, ra–right atrium, rv–right ventricle.
(TIFF)

S3 Fig *Hand1*^*LV*^*-Cre; R26R*^*lacZ/DTA*^ mice survive and do not exhibit abnormal cardiac function. A) *Hand1*^*LV*^*-Cre; R26R*^*lacZ/DTA*^ pups are recovered with expected Mendelian distribution at P28. B, C) Bisected P56 *Hand1*^*LV*^*-Cre(+); R26R*^*lacZ/DTA*^ hearts (B) display no gross structural abnormalities compared to control, *(-); R26R*^*lacZ/DTA*^ hearts (C). D-O) Echocardiography of these mice at P56 revealed no significant difference in echocardiographic parameters between *Hand1*^*LV*^*-Cre; R26R*^*lacZ/DTA*^ (n = 12) and control littermates (n = 8). d–diastole, s–systole, C–corrected, ID–internal diameter, PW–posterior wall.
(TIFF)

S4 Fig Hand gene expression at E11.5. In situ hybridization of E11.5 hearts to detect *Hand1* (A, B) and *Hand2* (C, D). *Hand1* cardiac expression is restricted to the LV myocardium, whereas *Hand2* at this stage of development is robustly expressed in the endocardium, epicardium and RV and LV myocardium.
(TIFF)

S5 Fig *Hand1*^*LV*^*-Cre*-mediated ablation of *Hand1* results in ventricular septal defects. A-O) H&E staining reveals that, by E17.5, *Hand1*^*LV*^*-Cre*;*Hand1*^*fx/fx*^*;Hand2*^*+/+*^ (G-I), *Hand1*^*LV*^*-Cre*;*Hand1*^*fx/fx*^*;Hand2*^*fx/+*^ (J-L), and *Hand1*^*LV*^*-Cre*;*Hand1*^*fx/fx*^*;Hand2*^*fx/fx*^ (M-O) hearts display ventricular septal defects (black arrowheads), an RV that communicates with both the pulmonary trunk and the aorta, and mitral valve hyperplasia. Asterisks denote the LV.
(TIFF)

S6 Fig Representative echocardiographs of DTA-rescued *Hand1*^*LV*^*-Cre Hand1*;*Hand2* conditional knockouts. A-F) B-mode (A-C) and M-mode (D-F) echocardiographic analyses of control (A, D), *Hand1*;*Hand2* CKO (B, E) and of *Hand1*;*Hand2* CKO DTA-rescued (C, F) mice at P56 reveals that the obstructive cardiomyocytes (E, yellow arrowhead) characteristic of *Hand1*^*LV*^*-Cre;Hand1*^*fx/fx*^*;Hand2*^*fx/fx*^ hearts are absent from *Hand1*^*LV*^*-Cre;Hand1*^*fx/fx*^;*Hand2*^*fx/fx*^;*R26R*^*+/eGFPDTA*^ hearts.
(TIFF)

S7 Fig Echocardiographic analyses of adult *Hand1*^*LV*^*-Cre*;*Hand1*;*Hand2* CKOs. A-J) Other than EF and FS, shown in Fig 4, echocardiography of *Hand1*^*LV*^*-Cre*;*Hand1*^*fx/+*^*;Hand2*^*fx/+*^, *Hand1*^*LV*^*-Cre*;*Hand1*^*fx/+*^*;Hand2*^*fx/fx*^, *Hand1*^*LV*^*-Cre*;*Hand1*^*fx/fx*^*;Hand2*^*fx/+*^, and *Hand1*^*LV*^*-Cre*;*Hand1*^*fx/fx*^*;Hand2*^*fx/fx*^ mice at P56 revealed no significant difference in additional echocardiographic parameters. Data are represented as mean ± standard error of mean. d–diastole, s–systole, C–corrected, ID–internal diameter, PW–posterior wall.
(TIFF)

S8 Fig *Hand1*^*LV*^*-Cre; R26R*^*lacZ/DTA*^ mice survive and do not exhibit abnormal cardiac function. A-C) Color photos of the X-gal-stained bisected P56 of *Control* (A), *Hand1*^*LV*^*-Cre*;*Hand1*^*fx/fx*^*;Hand2*^*fx/fx*^ (B), and *Hand1*^*LV*^*-Cre*;*Hand1*^*fx/fx*^*;Hand2*^*fx/fx*^;*R26R*^*DTA*^-rescued hearts (C) shown in Fig 7D) Section of an X-gal stained *Hand1*^*LV*^*-Cre*;*Hand1*^*fx/fx*^*;Hand2*^*fx/fx*^;*R26R*^*DTA*^-rescued heart showing persistent, lacZ-positive cells. E-J) Echocardiography of these mice at P56 revealed no significant difference in additional echocardiographic parameters between *Hand1*^*LV*^*-Cre(-);R26R*^*DTA*^ controls, denoted as DTA(+), *Hand1*^*LV*^*-Cre(+);Hand1*^*fx/fx*^*;Hand2*^*fx/fx*^ CKOs, and *Hand1*^*LV*^*-Cre(+);Hand1*^*fx/fx*^*;Hand2*^*fx/fx*^*;R26R*^*DTA*^ rescue mice. Data are represented as mean ± standard error of mean. d–diastole, s–systole, ID–internal diameter.
(TIFF)

S1 Dataset Figs 2C, 2D, 2U and 4 and 7M and 7N apply statistical evaluation of the data. The supplemental raw data speed sheet (excel file) presents the data and calculations for these figures in separate tabs. Fig 2C and 2D tabs show sections that are counterstained with propidium iodide (Quantification of the number of TUNEL-positive cells per heart in control and *Hand1LV-Cre(+);R26RlacZ/DTA* embryos at E9.5 (C) and E10.5 (D) were performed and Data are represented as mean ± standard error of mean. Asterisks denote significance (p ≤ 0.05) as determined by student’s t-test. Fig 2U tab shows) Quantification of pHH3+ cells relative to the number of DAPI+ pixels show that proliferation is not altered at E12.5, but is elevated specifically within the LV at E14.5. Data are represented as mean ± standard error of mean. Asterisks denote significance (p ≤ 0.05) as determined by student’s t-test. Fig 4 tab shows evaluation of mice at P56 reveal significant decreases in fractional shortening (A) and ejection fraction (B) in *Hand1LV-Cre*;*Hand1fx/fx;Hand2fx/fx* mutants relative to *Hand1LV-Cre*;*Hand1fx/+;Hand2fx/+* littermates. Data are represented as mean ± standard error of mean. Asterisks denote significance (p ≤ 0.05) as determined by student’s t-test. Fig 7M and 7N tab shows calculations for the functional analysis (EF and FS) of DTA ablation rescue in Fig 7. Data are represented as mean ± standard error of mean.
(XLSX)

## References

[1] Xin M, Olson EN, Bassel-Duby R. Mending broken hearts: cardiac development as a basis for adult heart regeneration and repair. Nat Rev Mol Cell Biol. 2013;14(8):529–41. doi: 10.1038/nrm3619 23839576 10.1038/nrm3619 PMC3757945

[2] Towbin JA, Lorts A, Jefferies JL. Left ventricular non-compaction cardiomyopathy. Lancet. 2015;386(9995):813–25. doi: 10.1016/S0140-6736(14)61282-4 25865865 10.1016/S0140-6736(14)61282-4

[3] Hussein A, Karimianpour A, Collier P, Krasuski RA. Isolated Noncompaction of the Left Ventricle in Adults. Journal of the American College of Cardiology. 2015;66(5):578–85. doi: 10.1016/j.jacc.2015.06.017 26227197 10.1016/j.jacc.2015.06.017

[4] Tchervenkov CI, Walters HL, 3rd, Chu VF. Congenital Heart Surgery Nomenclature and Database Project: double outlet left ventricle. Ann Thorac Surg. 2000;69(4 Suppl):S264–9. 10798434 10.1016/s0003-4975(99)01281-3

[5] Gordon BM, Rodriguez S, Lee M, Chang RK. Decreasing number of deaths of infants with hypoplastic left heart syndrome. J Pediatr. 2008;153(3):354–8. doi: 10.1016/j.jpeds.2008.03.009 18534240 10.1016/j.jpeds.2008.03.009

[6] Rochais F, Mesbah K, Kelly RG. Signaling pathways controlling second heart field development. Circ Res. 2009;104(8):933–42. doi: 10.1161/CIRCRESAHA.109.194464 19390062 10.1161/CIRCRESAHA.109.194464

[7] Barnes RM, Firulli BA, Conway SJ, Vincentz JW, Firulli AB. Analysis of the Hand1 cell lineage reveals novel contributions to cardiovascular, neural crest, extra-embryonic, and lateral mesoderm derivatives. Developmental dynamics: an official publication of the American Association of Anatomists. 2010;239(11):3086–97. 20882677 10.1002/dvdy.22428 PMC2965316

[8] Reamon-Buettner SM, Ciribilli Y, Inga A, Borlak J. A loss-of-function mutation in the binding domain of HAND1 predicts hypoplasia of the human hearts. Human molecular genetics. 2008;17(10):1397–405. doi: 10.1093/hmg/ddn027 18276607 10.1093/hmg/ddn027

[9] McFadden DG, Barbosa AC, Richardson JA, Schneider MD, Srivastava D, Olson EN. The Hand1 and Hand2 transcription factors regulate expansion of the embryonic cardiac ventricles in a gene dosage-dependent manner. Development. 2005;132(1):189–201. doi: 10.1242/dev.01562 15576406 10.1242/dev.01562

[10] Barnes RM, Firulli BA, VanDusen NJ, Morikawa Y, Conway SJ, Cserjesi P, et al Hand2 loss-of-function in Hand1-expressing cells reveals distinct roles in epicardial and coronary vessel development. Circulation research. 2011;108(8):940–9. doi: 10.1161/CIRCRESAHA.110.233171 21350214 10.1161/CIRCRESAHA.110.233171 PMC3086599

[11] Zaruba MM, Soonpaa M, Reuter S, Field LJ. Cardiomyogenic potential of C-kit(+)-expressing cells derived from neonatal and adult mouse hearts. Circulation. 2010;121(18):1992–2000. doi: 10.1161/CIRCULATIONAHA.109.909093 20421520 10.1161/CIRCULATIONAHA.109.909093 PMC2879145

[12] Maillet M, van Berlo JH, Molkentin JD. Molecular basis of physiological heart growth: fundamental concepts and new players. Nat Rev Mol Cell Biol. 2013;14(1):38–48. doi: 10.1038/nrm3495 23258295 10.1038/nrm3495 PMC4416212

[13] Reuter S, Soonpaa MH, Firulli AB, Chang AN, Field LJ. Recombinant neuregulin 1 does not activate cardiomyocyte DNA synthesis in normal or infarcted adult mice. PLoS One. 2014;9(12):e115871 doi: 10.1371/journal.pone.0115871 25545368 10.1371/journal.pone.0115871 PMC4278834

[14] Porrello ER, Mahmoud AI, Simpson E, Hill JA, Richardson JA, Olson EN, et al Transient regenerative potential of the neonatal mouse heart. Science. 2011;331(6020):1078–80. doi: 10.1126/science.1200708 21350179 10.1126/science.1200708 PMC3099478

[15] Drenckhahn JD, Schwarz QP, Gray S, Laskowski A, Kiriazis H, Ming Z, et al Compensatory growth of healthy cardiac cells in the presence of diseased cells restores tissue homeostasis during heart development. Developmental cell. 2008;15(4):521–33. doi: 10.1016/j.devcel.2008.09.005 18854137 10.1016/j.devcel.2008.09.005

[16] Sturzu AC, Rajarajan K, Passer D, Plonowska K, Riley A, Tan TC, et al Fetal Mammalian Heart Generates a Robust Compensatory Response to Cell Loss. Circulation. 2015;132(2):109–21. doi: 10.1161/CIRCULATIONAHA.114.011490 25995316 10.1161/CIRCULATIONAHA.114.011490 PMC4516129

[17] Vinhas M, Araujo AC, Ribeiro S, Rosario LB, Belo JA. Transthoracic echocardiography reference values in juvenile and adult 129/Sv mice. Cardiovasc Ultrasound. 2013;11:12 doi: 10.1186/1476-7120-11-12 23634975 10.1186/1476-7120-11-12 PMC3651272

[18] Tsuchihashi T, Maeda J, Shin CH, Ivey KN, Black BL, Olson EN, et al Hand2 function in second heart field progenitors is essential for cardiogenesis. Developmental biology. 2011;351(1):62–9. doi: 10.1016/j.ydbio.2010.12.023 21185281 10.1016/j.ydbio.2010.12.023 PMC3039109

[19] VanDusen NJ, Casanovas J, Vincentz JW, Firulli BA, Osterwalder M, Lopez-Rios J, et al Hand2 is an essential regulator for two Notch-dependent functions within the embryonic endocardium. Cell reports. 2014;9(6):2071–83. doi: 10.1016/j.celrep.2014.11.021 25497097 10.1016/j.celrep.2014.11.021 PMC4277501

[20] Koibuchi N, Chin MT. CHF1/Hey2 plays a pivotal role in left ventricular maturation through suppression of ectopic atrial gene expression. Circ Res. 2007;100(6):850–5. doi: 10.1161/01.RES.0000261693.13269.bf 17332425 10.1161/01.RES.0000261693.13269.bf

[21] Singh MK, Christoffels VM, Dias JM, Trowe MO, Petry M, Schuster-Gossler K, et al Tbx20 is essential for cardiac chamber differentiation and repression of Tbx2. Development. 2005;132(12):2697–707. doi: 10.1242/dev.01854 15901664 10.1242/dev.01854

[22] Sizarov A, Ya J, de Boer BA, Lamers WH, Christoffels VM, Moorman AF. Formation of the building plan of the human heart: morphogenesis, growth, and differentiation. Circulation. 2011;123(10):1125–35. doi: 10.1161/CIRCULATIONAHA.110.980607 21403123 10.1161/CIRCULATIONAHA.110.980607

[23] Scholzen T, Gerdes J. The Ki-67 protein: from the known and the unknown. J Cell Physiol. 2000;182(3):311–22. doi: 10.1002/(SICI)1097-4652(200003)182:3<311::AID-JCP1>3.0.CO;2-9 10653597 10.1002/(SICI)1097-4652(200003)182:3<311::AID-JCP1>3.0.CO;2-9

[24] Togi K, Kawamoto T, Yamauchi R, Yoshida Y, Kita T, Tanaka M. Role of Hand1/eHAND in the dorso-ventral patterning and interventricular septum formation in the embryonic heart. Mol Cell Biol. 2004;24(11):4627–35. doi: 10.1128/MCB.24.11.4627-4635.2004 15143159 10.1128/MCB.24.11.4627-4635.2004 PMC416422

[25] Koshiba-Takeuchi K, Mori AD, Kaynak BL, Cebra-Thomas J, Sukonnik T, Georges RO, et al Reptilian heart development and the molecular basis of cardiac chamber evolution. Nature. 2009;461(7260):95–8. doi: 10.1038/nature08324 19727199 10.1038/nature08324 PMC2753965

[26] Oechslin EN, Attenhofer Jost CH, Rojas JR, Kaufmann PA, Jenni R. Long-term follow-up of 34 adults with isolated left ventricular noncompaction: a distinct cardiomyopathy with poor prognosis. Journal of the American College of Cardiology. 2000;36(2):493–500. 10933363 10.1016/s0735-1097(00)00755-5

[27] Cserjesi P, Brown D, Lyons GE, Olson EN. Expression of the novel basic helix-loop-helix gene eHAND in neural crest derivatives and extraembryonic membranes during mouse development. Developmental biology. 1995;170(2):664–78. doi: 10.1006/dbio.1995.1245 7649392 10.1006/dbio.1995.1245

[28] Cai CL, Liang X, Shi Y, Chu PH, Pfaff SL, Chen J, et al Isl1 identifies a cardiac progenitor population that proliferates prior to differentiation and contributes a majority of cells to the heart. Developmental cell. 2003;5(6):877–89. 14667410 10.1016/s1534-5807(03)00363-0 PMC5578462

[29] Sun Y, Liang X, Najafi N, Cass M, Lin L, Cai CL, et al Islet 1 is expressed in distinct cardiovascular lineages, including pacemaker and coronary vascular cells. Developmental biology. 2007;304(1):286–96. doi: 10.1016/j.ydbio.2006.12.048 17258700 10.1016/j.ydbio.2006.12.048 PMC2582044

[30] Moretti A, Caron L, Nakano A, Lam JT, Bernshausen A, Chen Y, et al Multipotent embryonic isl1+ progenitor cells lead to cardiac, smooth muscle, and endothelial cell diversification. Cell. 2006;127(6):1151–65. doi: 10.1016/j.cell.2006.10.029 17123592 10.1016/j.cell.2006.10.029

[31] Milgrom-Hoffman M, Harrelson Z, Ferrara N, Zelzer E, Evans SM, Tzahor E. The heart endocardium is derived from vascular endothelial progenitors. Development. 2011;138(21):4777–87. doi: 10.1242/dev.061192 21989917 10.1242/dev.061192 PMC3190386

[32] Jensen B, van der Wal AC, Moorman AF, Christoffels VM. Excessive trabeculations in noncompaction do not have the embryonic identity. Int J Cardiol. 2017;227:325–30. doi: 10.1016/j.ijcard.2016.11.089 27838129 10.1016/j.ijcard.2016.11.089

[33] Liu X, Yagi H, Saeed S, Bais AS, Gabriel GC, Chen Z, et al The complex genetics of hypoplastic left heart syndrome. Nature genetics. 2017;49(7):1152–9. doi: 10.1038/ng.3870 28530678 10.1038/ng.3870 PMC5737968

[34] Barbosa AC, Funato N, Chapman S, McKee MD, Richardson JA, Olson EN, et al Hand transcription factors cooperatively regulate development of the distal midline mesenchyme. Developmental biology. 2007;310(1):154–68. doi: 10.1016/j.ydbio.2007.07.036 17764670 10.1016/j.ydbio.2007.07.036 PMC2270479

[35] Ivanova A, Signore M, Caro N, Greene ND, Copp AJ, Martinez-Barbera JP. In vivo genetic ablation by Cre-mediated expression of diphtheria toxin fragment A. Genesis. 2005;43(3):129–35. doi: 10.1002/gene.20162 16267821 10.1002/gene.20162 PMC2233880

[36] Soriano P. Generalized lacZ expression with the ROSA26 Cre reporter strain. Nature genetics. 1999;21(1):70–1. doi: 10.1038/5007 9916792 10.1038/5007

[37] Firulli AB, McFadden DG, Lin Q, Srivastava D, Olson EN. Heart and extra-embryonic mesodermal defects in mouse embryos lacking the bHLH transcription factor Hand1. Nature genetics. 1998;18(3):266–70. doi: 10.1038/ng0398-266 9500550 10.1038/ng0398-266

[38] Vincentz JW, VanDusen NJ, Fleming AB, Rubart M, Firulli BA, Howard MJ, et al A Phox2- and Hand2-dependent Hand1 cis-regulatory element reveals a unique gene dosage requirement for Hand2 during sympathetic neurogenesis. The Journal of neuroscience: the official journal of the Society for Neuroscience. 2012;32(6):2110–20. 22323723 10.1523/JNEUROSCI.3584-11.2012 PMC3324095

[39] Firulli BA, Fuchs RK, Vincentz JW, Clouthier DE, Firulli AB. Hand1 phosphoregulation within the distal arch neural crest is essential for craniofacial morphogenesis. Development. 2014;141(15):3050–61. doi: 10.1242/dev.107680 25053435 10.1242/dev.107680 PMC4197675

[40] Vincentz JW, Firulli BA, Lin A, Spicer DB, Howard MJ, Firulli AB. Twist1 controls a cell-specification switch governing cell fate decisions within the cardiac neural crest. PLoS genetics. 2013;9(3):e1003405 doi: 10.1371/journal.pgen.1003405 23555309 10.1371/journal.pgen.1003405 PMC3605159

[41] Vincentz JW, Barnes RM, Firulli BA, Conway SJ, Firulli AB. Cooperative interaction of Nkx2.5 and Mef2c transcription factors during heart development. Developmental dynamics: an official publication of the American Association of Anatomists. 2008;237(12):3809–19. 19035347 10.1002/dvdy.21803 PMC2639719

[42] Vincentz JW, Casasnovas JJ, Barnes RM, Que J, Clouthier DE, Wang J, et al Exclusion of Dlx5/6 expression from the distal-most mandibular arches enables BMP-mediated specification of the distal cap. Proceedings of the National Academy of Sciences of the United States of America. 2016;113(27):7563–8. doi: 10.1073/pnas.1603930113 27335460 10.1073/pnas.1603930113 PMC4941460

[43] Schiller NB, Shah PM, Crawford M, DeMaria A, Devereux R, Feigenbaum H, et al Recommendations for quantitation of the left ventricle by two-dimensional echocardiography. American Society of Echocardiography Committee on Standards, Subcommittee on Quantitation of Two-Dimensional Echocardiograms. J Am Soc Echocardiogr. 1989;2(5):358–67. 2698218 10.1016/s0894-7317(89)80014-8

