# Supplementary figures and images for: Hand factor ablation causes defective left ventricular chamber development and compromised adult cardiac function
Source: PLoS Genet. 2017 Jul 21;13(7):e1006922. doi: 10.1371/journal.pgen.1006922 (PMC5544250; doi:10.1371/journal.pgen.1006922)

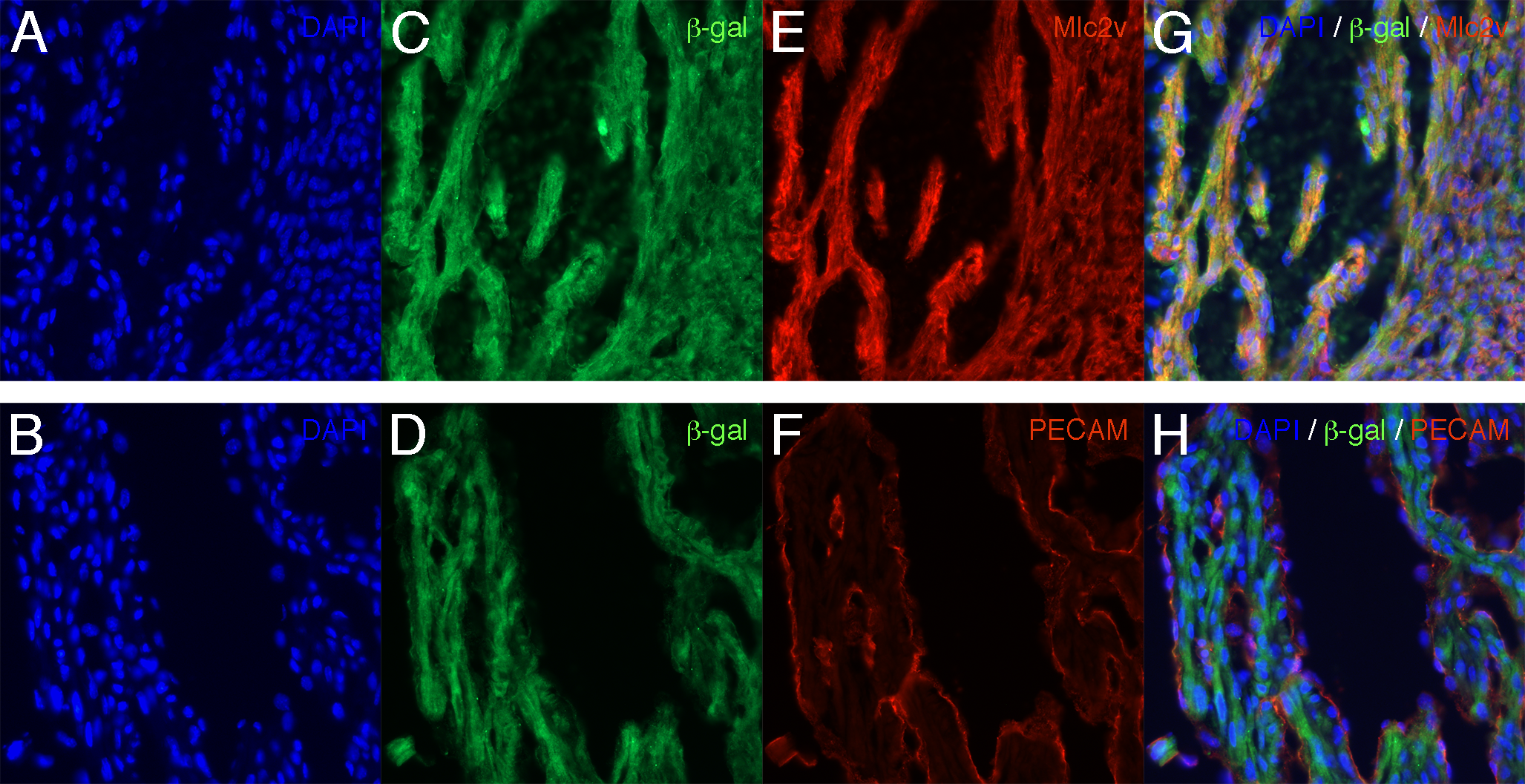

Supplement: S1 Fig — A-H) Immunohistochemistry on E15.5 Hand1LV-Cre(+);R26R+/lacZ hearts for β-galactosidase (C, D), Mlc2v (E), and PECAM (F) show that the Hand1LV-Cre lineage is predominantly myocardial (yellow co-localization in G), and not endocardial (H). The apical free wall of the LV is shown. Sections are counterstained with DAPI (A, B). (TIFF) [file pgen.1006922.s001.tiff]

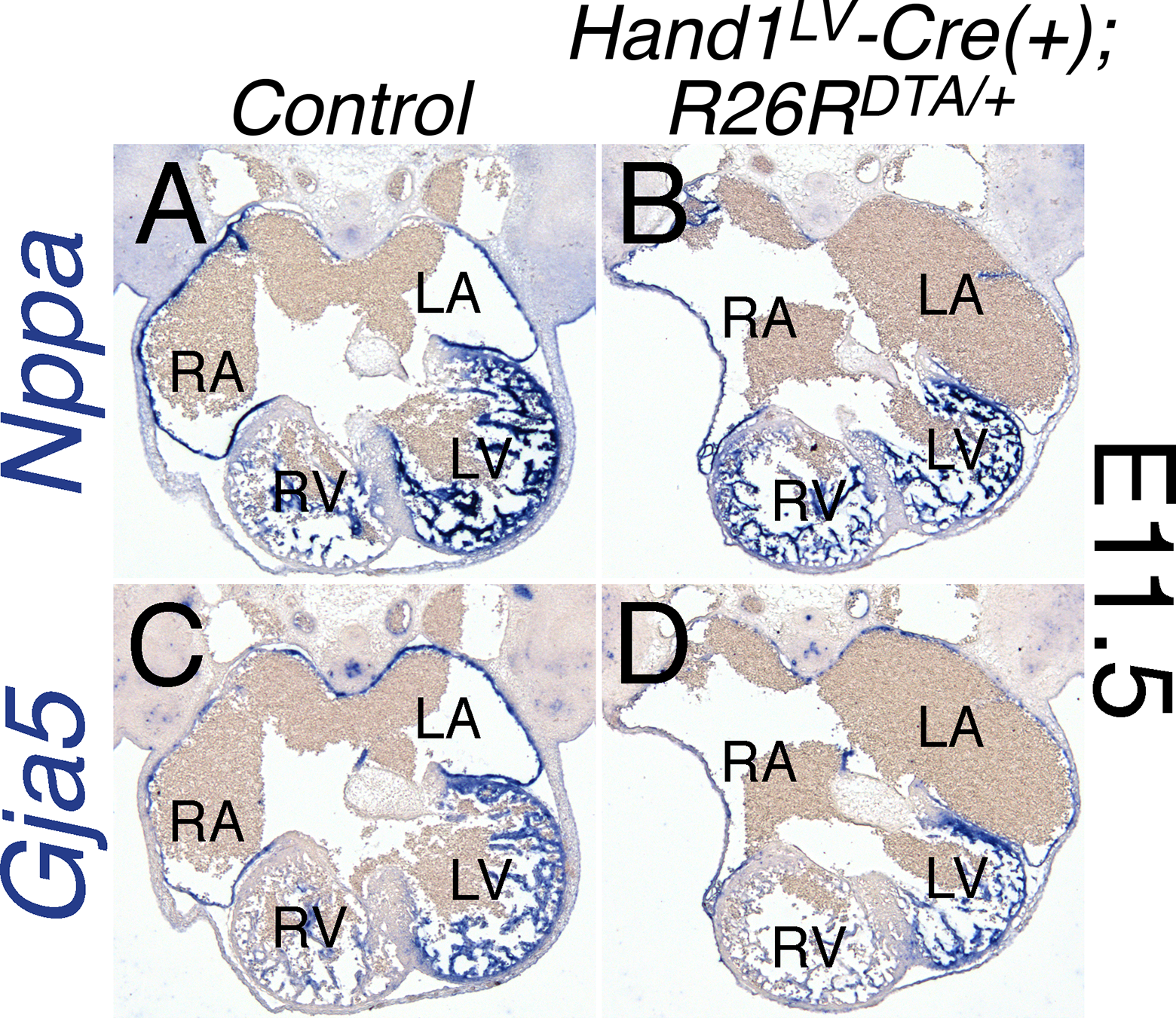

Supplement: S2 Fig — A-D) Section in situ hybridization of E11.5 hearts showing expression of LV markers Nppa (A, B) and Gja5 (C, D). la–left atrium, lv–left ventricle, ra–right atrium, rv–right ventricle. (TIFF) [file pgen.1006922.s002.tiff]

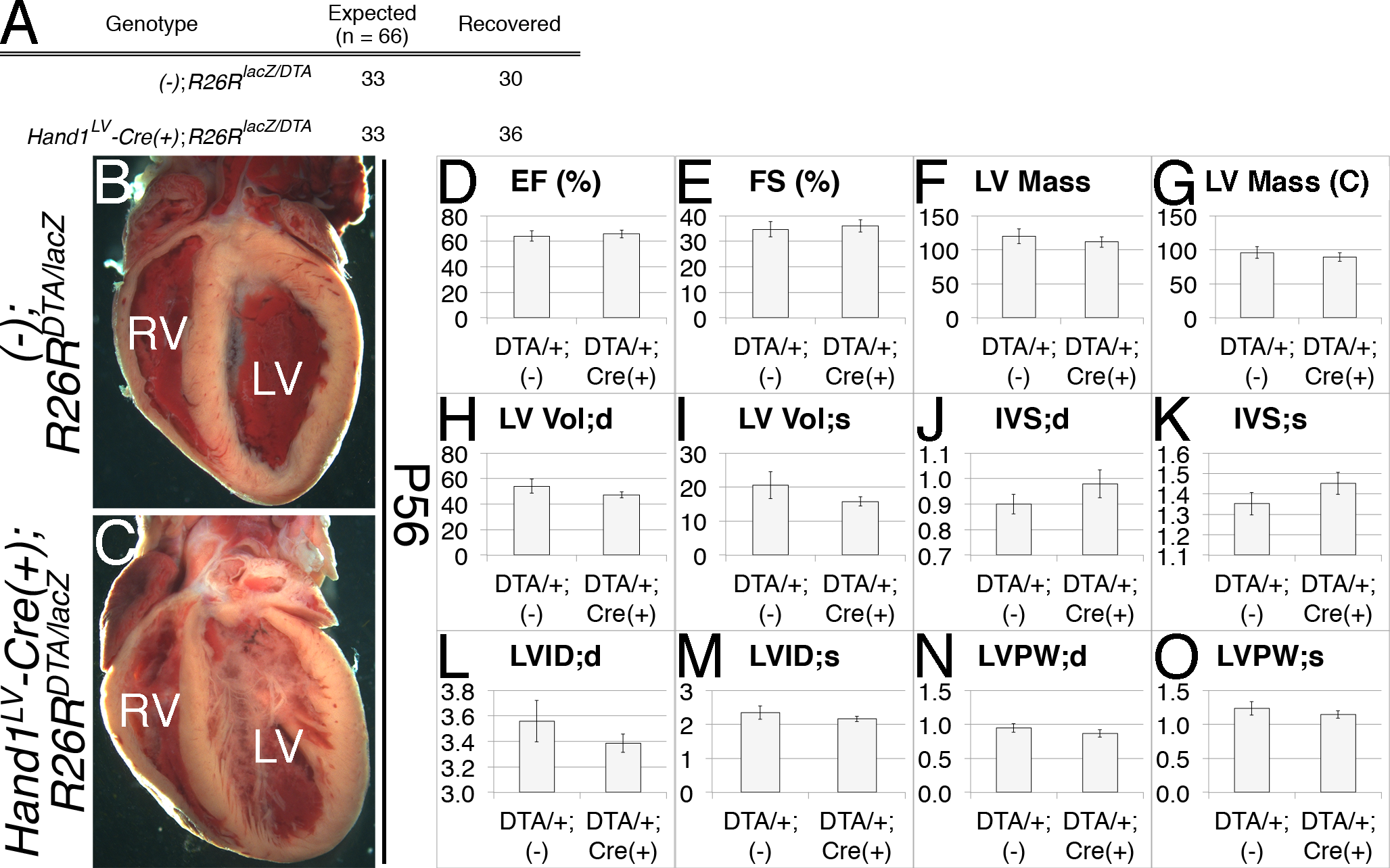

Supplement: S3 Fig — A) Hand1LV-Cre; R26RlacZ/DTA pups are recovered with expected Mendelian distribution at P28. B, C) Bisected P56 Hand1LV-Cre(+); R26RlacZ/DTA hearts (B) display no gross structural abnormalities compared to control, (-); R26RlacZ/DTA hearts (C). D-O) Echocardiography of these mice at P56 revealed no significant difference in echocardiographic parameters between Hand1LV-Cre; R26RlacZ/DTA (n = 12) and control littermates (n = 8). d–diastole, s–systole, C–corrected, ID–internal diameter, PW–posterior wall. (TIFF) [file pgen.1006922.s003.tiff]

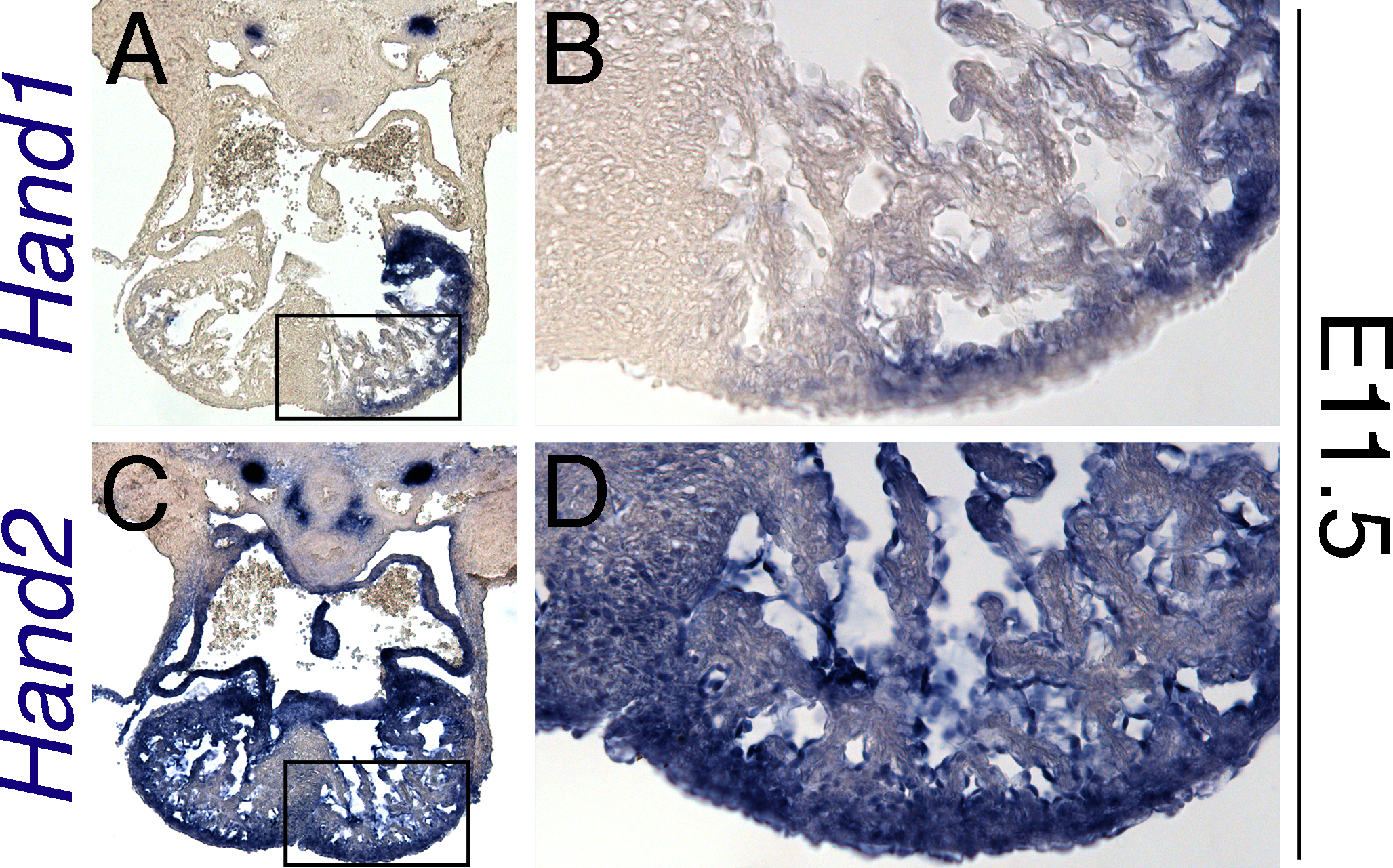

Supplement: S4 Fig — In situ hybridization of E11.5 hearts to detect Hand1 (A, B) and Hand2 (C, D). Hand1 cardiac expression is restricted to the LV myocardium, whereas Hand2 at this stage of development is robustly expressed in the endocardium, epicardium and RV and LV myocardium. (TIFF) [file pgen.1006922.s004.tiff]

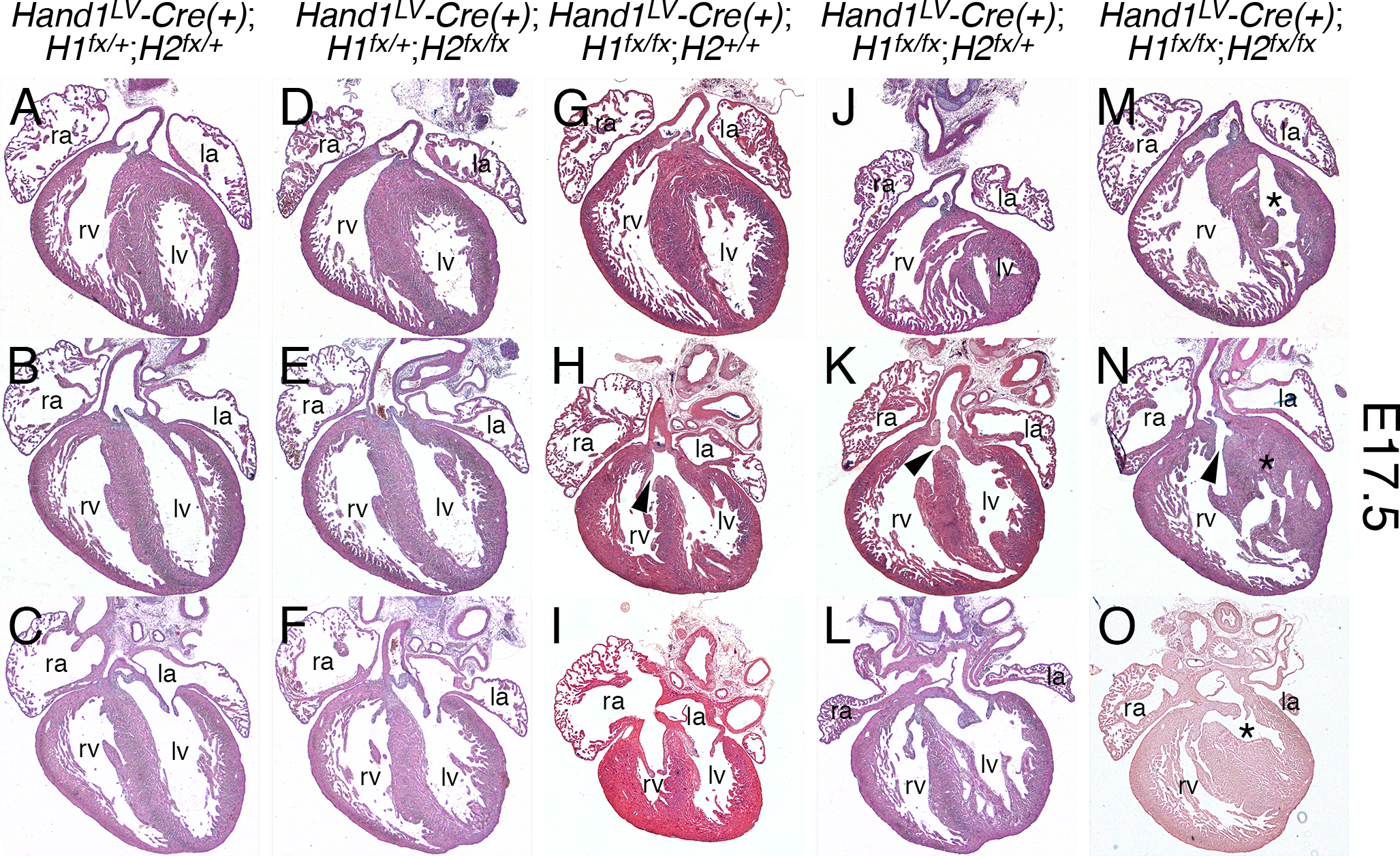

Supplement: S5 Fig — A-O) H&E staining reveals that, by E17.5, Hand1LV-Cre;Hand1fx/fx;Hand2+/+ (G-I), Hand1LV-Cre;Hand1fx/fx;Hand2fx/+ (J-L), and Hand1LV-Cre;Hand1fx/fx;Hand2fx/fx (M-O) hearts display ventricular septal defects (black arrowheads), an RV that communicates with both the pulmonary trunk and the aorta, and mitral valve hyperplasia. Asterisks denote the LV. (TIFF) [file pgen.1006922.s005.tiff]

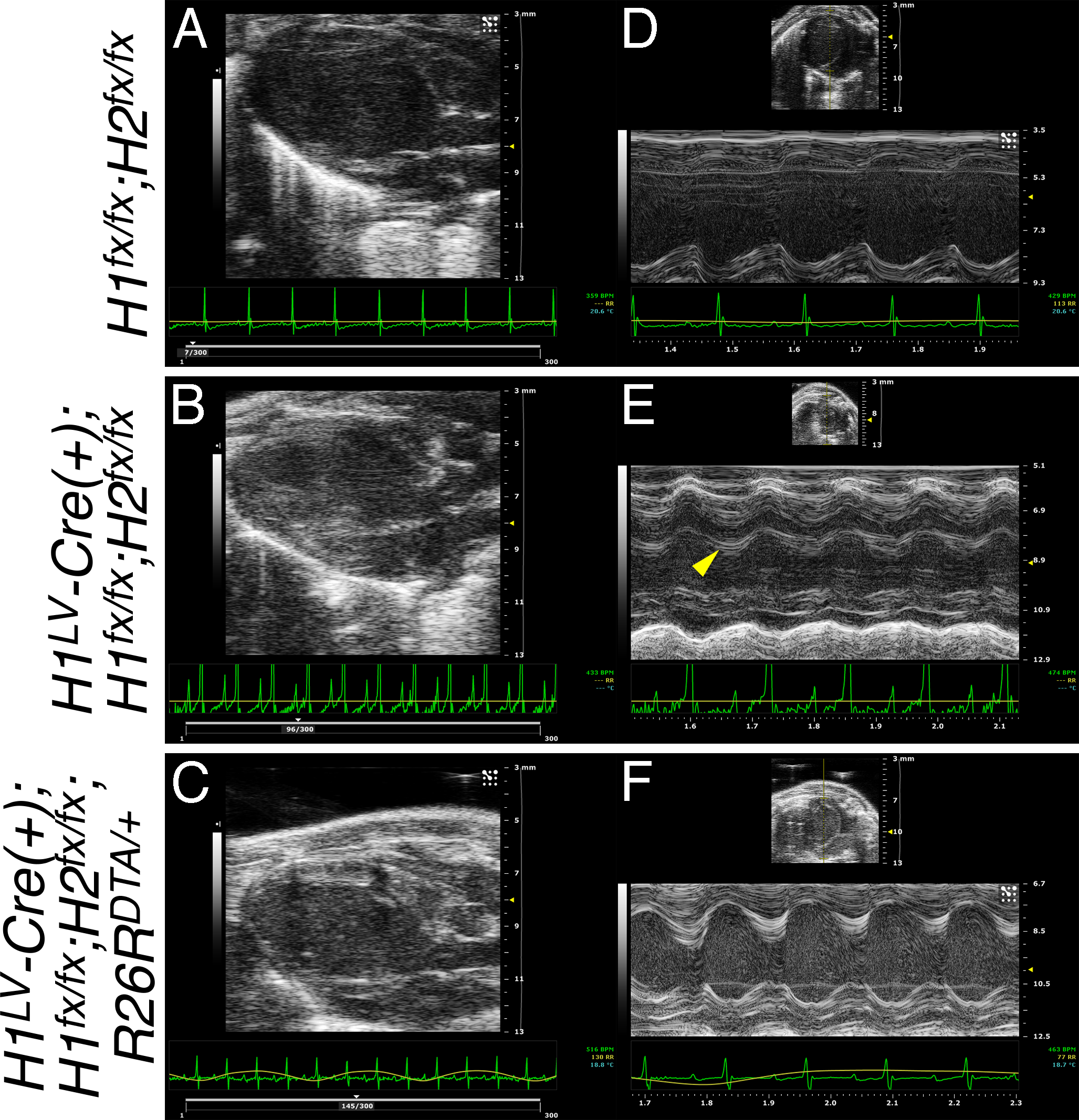

Supplement: S6 Fig — A-F) B-mode (A-C) and M-mode (D-F) echocardiographic analyses of control (A, D), Hand1;Hand2 CKO (B, E) and of Hand1;Hand2 CKO DTA-rescued (C, F) mice at P56 reveals that the obstructive cardiomyocytes (E, yellow arrowhead) characteristic of Hand1LV-Cre;Hand1fx/fx;Hand2fx/fx hearts are absent from Hand1LV-Cre;Hand1fx/fx;Hand2fx/fx;R26R+/eGFPDTA hearts. (TIFF) [file pgen.1006922.s006.tiff]

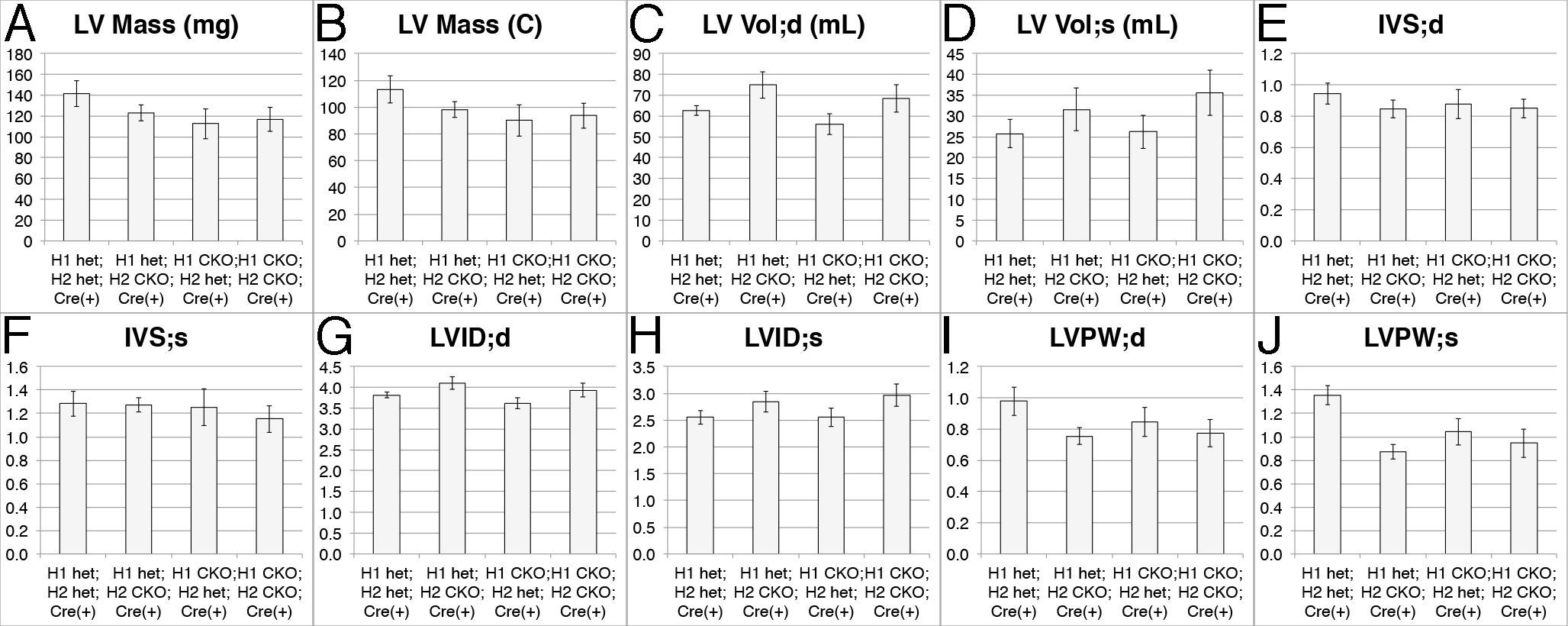

Supplement: S7 Fig — A-J) Other than EF and FS, shown in Fig 4, echocardiography of Hand1LV-Cre;Hand1fx/+;Hand2fx/+, Hand1LV-Cre;Hand1fx/+;Hand2fx/fx, Hand1LV-Cre;Hand1fx/fx;Hand2fx/+, and Hand1LV-Cre;Hand1fx/fx;Hand2fx/fx mice at P56 revealed no significant difference in additional echocardiographic parameters. Data are represented as mean ± standard error of mean. d–diastole, s–systole, C–corrected, ID–internal diameter, PW–posterior wall. (TIFF) [file pgen.1006922.s007.tiff]

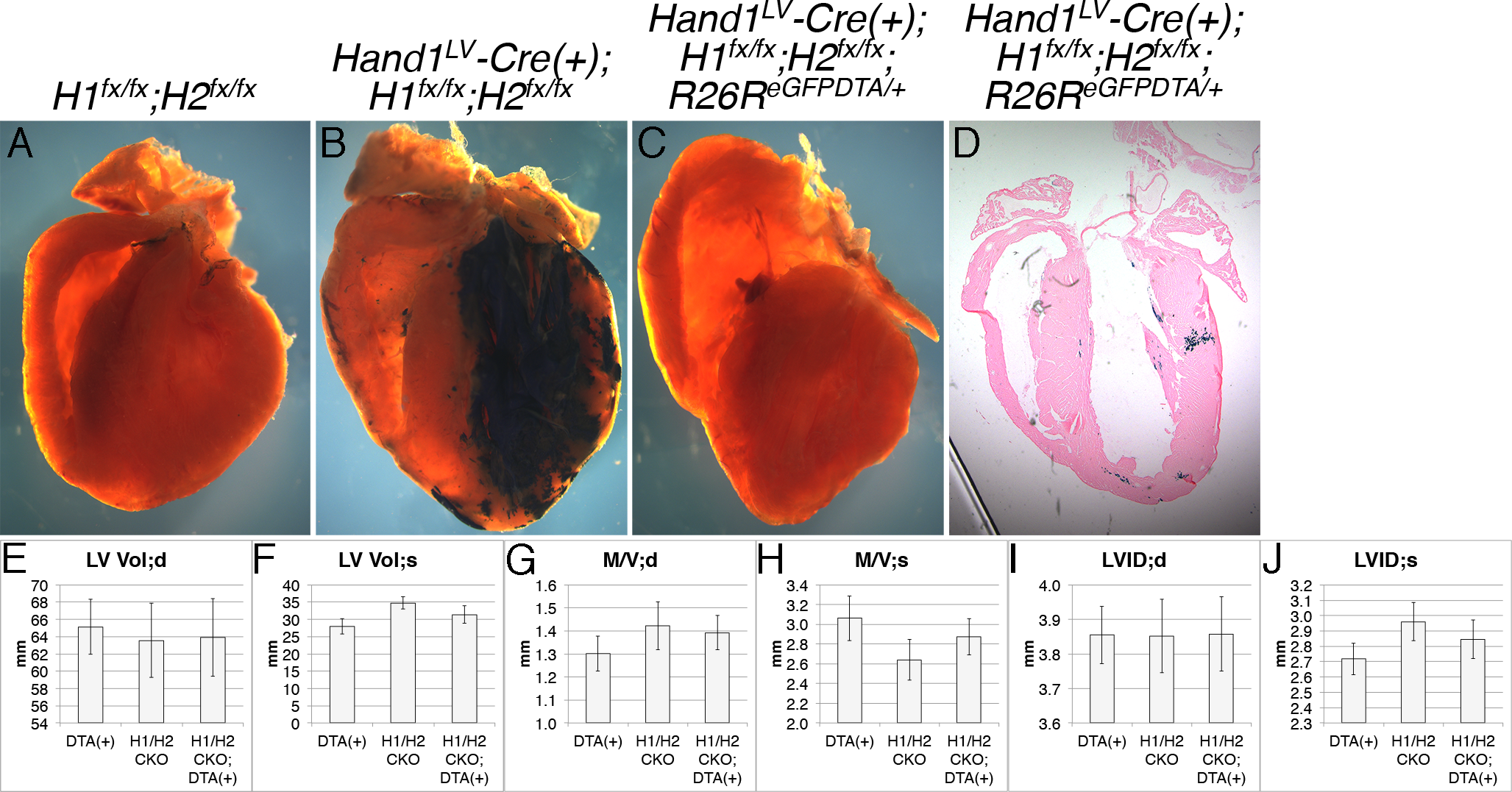

Supplement: S8 Fig — A-C) Color photos of the X-gal-stained bisected P56 of Control (A), Hand1LV-Cre;Hand1fx/fx;Hand2fx/fx (B), and Hand1LV-Cre;Hand1fx/fx;Hand2fx/fx;R26RDTA-rescued hearts (C) shown in Fig 7D) Section of an X-gal stained Hand1LV-Cre;Hand1fx/fx;Hand2fx/fx;R26RDTA-rescued heart showing persistent, lacZ-positive cells. E-J) Echocardiography of these mice at P56 revealed no significant difference in additional echocardiographic parameters between Hand1LV-Cre(-);R26RDTA controls, denoted as DTA(+), Hand1LV-Cre(+);Hand1fx/fx;Hand2fx/fx CKOs, and Hand1LV-Cre(+);Hand1fx/fx;Hand2fx/fx;R26RDTA rescue mice. Data are represented as mean ± standard error of mean. d–diastole, s–systole, ID–internal diameter. (TIFF) [file pgen.1006922.s008.tiff]
